# Continuously Quantifying Oral Chemicals Based on Flexible Hybrid Electronics for Clinical Diagnosis and Pathogenetic Study

**DOI:** 10.34133/2022/9810129

**Published:** 2022-08-16

**Authors:** Wei Ling, Yinghui Wang, Bingyu Lu, Xue Shang, Ziyue Wu, Zhaorun Chen, Xueting Li, Chenchen Zou, Jinjie Yan, Yunjie Zhou, Jie Liu, Hongjie Li, Kehua Que, Xian Huang

**Affiliations:** ^1^Department of Biomedical Engineering, Tianjin University, 92 Weijin Road, Tianjin 300072, China; ^2^Center of Flexible Wearable Technology, Institute of Flexible Electronic Technology of Tsinghua, Jiaxing 314006, China; ^3^School of Stomatology, Hospital of Stomatology, Tianjin Medical University, 12 Observatory Road, Tianjin 300070, China

## Abstract

Simultaneous monitoring of diverse salivary parameters can reveal underlying mechanisms of intraoral biological processes and offer profound insights into the evolution of oral diseases. However, conventional analytical devices with bulky volumes, rigid formats, and discrete sensing mechanisms deviate from the requirements of continuous biophysiological quantification, resulting in huge difficulty in precise clinical diagnosis and pathogenetic study. Here, we present a flexible hybrid electronic system integrated with functional nanomaterials to continuously sense Ca^2+^, pH, and temperature for wireless real-time oral health monitoring. The miniaturized system with an island-bridge structure that is designed specifically to fit the teeth is only 0.4 g in weight and 31.5 × 8.5 × 1.35 mm^3^ in dimension, allowing effective integration with customized dental braces and comfort attachment on teeth. Characterization results indicate high sensitivities of 30.3 and 60.6 mV/decade for Ca^2+^ and pH with low potential drifts. The system has been applied in clinical studies to conduct Ca^2+^ and pH mappings on carious teeth, biophysiological monitoring for up to 12 h, and outcome evaluation of dental restoration, providing quantitative data to assist in the diagnosis and understanding of oral diseases. Notably, caries risk assessment of 10 human subjects using the flexible system validates the important role of saliva buffering capacity in caries pathogenesis. The proposed flexible system may offer an open platform to carry diverse components to support both clinical diagnosis and treatment as well as fundamental research for oral diseases and induced systemic diseases.

## 1. Introduction

Irreversible pathological loss in enamel and dentin due to acid-induced dental erosion and caries is rampant with a global prevalence of ~40% [1, 2]. Such oral diseases can lead to foul breath, toothache, and even chronic systemic infections at escalated stages [2, 3]. The seriousness of these diseases has long been underestimated, especially in many underdeveloped and developing countries. Despite multiple hypotheses about the etiology of such diseases [4, 5], the underlying pathogenesis and evolutionary process have not been fully revealed due to the lack of tools for continuously quantifying the physiological processes. Saliva contains complex compositions that include microorganisms, electrolytes, proteins, and peptides, all of which may be used as indicators for diagnosing oral and systemic diseases [6, 7]. Despite the significant roles of microorganisms in oral disease, direct detection of these microorganisms *in vivo* may not be feasible, as it may require highly specific biological indicators that may not be biocompatible to the oral environment and complex sensing mechanisms that are difficult to achieve using fully integrated and miniaturized devices [8–10]. Since many literatures have suggested that salivary electrolytes such as Ca^2+^ and pH are related to multiple oral diseases [11, 12], continuous monitoring of these two indicators may provide rich *in vivo* data for the diagnosis and etiological analysis of oral diseases. However, current diagnostic and analytical tools that conduct imaging (e.g., X-ray and CT), *in vivo* monitoring (e.g., pulp vitality testing), and *in vitro* analysis (e.g., saliva sampling) are unable to continuously collect intraoral biological parameters due to their discrete working mechanisms. The rigid and bulky formats of these tools are mechanically incompatible with the soft and curvilinear oral tissues, leading to poor user experience and obstacles to performing long-term continuous monitoring. Despite the urgent demands of quantitative data for oral disease, a fully integrated system for long-term continuous physiological monitoring for teeth has seldomly been realized.

Emerging biointegrated flexible electronics feature conformal and intimate contact to the soft and curved tissues with numerous demonstrations in sensing physiological signals such as temperature [13], pressure [14], heart rate [15], and biopotential [16], as well as chemicals such as glucose [17], lactate [18], cortisol [19], neurotransmitters [20], and electrolytes [21]. Such flexible devices capable of detecting various vital signals have been conformally integrated on the skin for simultaneous and selective monitoring of a panel of biomarkers in sweat [22, 23]. Similar techniques may be adopted to develop wearable devices that possess superior adaptability to teeth with low profiles and low modulus. Despite multiple published devices that have presented great potential [24–27], the majority of them are either based on pure physical sensing or only contain partially flexible components connected with rigid external circuits. A flexible system with fully integrated sensing, processing, communicating, and power-supplying functions has rarely been reported. Integration of flexible devices on teeth is quite different from direct skin surface mounting and complete body implantation, as the device is noninvasively attached to tissue in the mouth, which is a semiclosed environment directly connected to internal digestion systems. The sensing results are subject to complicated influences from saliva composition, food intake, varied pH values, and motion artifacts, all of which may cause sensor degradation, biomolecule interference, and signal fluctuation. In addition, practical issues such as wireless transmission, power supply, device fixture, and biocompatibility packaging in wet and narrow spaces also demand thorough exploration to accommodate stringent clinic requirements.

Here, we present a low-profile, modularized multifunctional hybrid electronic system for oral health monitoring and investigate techniques to achieve stable electrochemical sensing in complex oral environments. The flexible system contains two electrochemical sensors for Ca^2+^ and pH sensing and a miniaturized wireless circuit for signal processing and transmission. Since electrochemical detection tends to be influenced by temperature fluctuation, the real-time oral temperature is also measured simultaneously as a calibration parameter for electrochemical sensors. The system, which is only 0.4 g in weight and 1.35 mm in height can be integrated with specifically customized dental braces, allowing conformal contact to the measuring position and comfortable wearing without influencing the daily activities of wearers. The flexible system can withstand temperature fluctuation and repeated external stress with small drifts of 1.4 mV/h. Modifications of functional sensing materials on the flexible sensors lead to high sensitivities of 30.3 and 60.6 mV/decade for Ca^2+^ and pH, respectively, while the surface modification and system package ensure a lifespan of more than 20 days, during which white spot lesions can develop as a result of plaque accumulation due to inadequate oral hygiene [28]. The system was first tested *in vitro* using artificial saliva and decayed teeth. The excellent biocompatibility demonstrated by cytotoxicity studies has enabled its clinic applications in continuous biophysiological monitoring for more than 12 h, evaluating the outcome of dental restoration, and assessing caries risk in 10 human subjects. The results that are consistent with clinical diagnosis demonstrate that the system can be used to assist diagnosis and guide dental care. Notably, the system offers quantitative data on the dynamics of salivary buffering and validates the association of saliva buffering capacity with caries risk, indicating promising applications in caries risk assessment and etiological analysis. The electrochemical sensors can also be modified with other biocompatible chemicals or biological substances to detect different molecules, enabling sophisticated applications in verifying clinical hypotheses. The proposed flexible system may offer an open platform to carry diverse sensing, stimulation, and treatment components that can support fundamental research for better understanding mechanisms of oral diseases and their functions in triggering systemic diseases, such as diabetes, Parkinson's, and oral cancer. In combination with artificial intelligence technology, the dynamic data collected by these systems may be used to predict the evolution of the diseases, allowing early disease prevention and more personalized treatment plans.

## 2. Results

### 2.1. Design and Fabrication of the Flexible Electronic System

**Figure 1:**
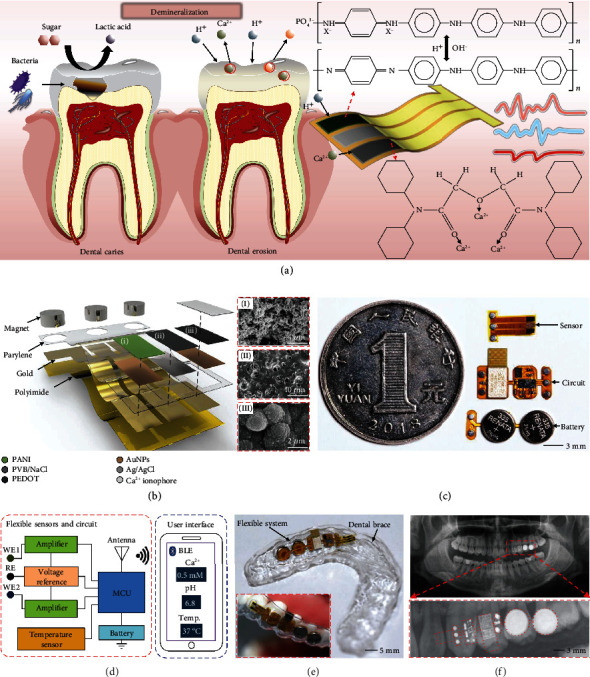
Modularized flexible electronic systems for real-time oral health monitoring. (a) A schematic of an implantable flexible system modified with functional materials for monitoring intraoral Ca^2+^, pH, and temperature simultaneously. (b) An exploded view of a sensor integrated with multiple functional nanomaterials. Insets: SEM images showing the surface morphology of the (I) PANI, (II) PVB/NaCl, and (III) PEDOT layers modified on corresponding electrodes. (c) An image of a modularized system that includes three electrochemical electrodes for chemical sensing, a flexible circuit for data transmission, and two batteries connected in series for power supply. (d) A system-level diagram showing the electrical components of the system. (e) A flexible dental brace embedded with a system for oral health monitoring. Inset: a flexible system fixed on teeth. (f) A panoramic X-ray image of an oral cavity showing the deployment of a system on teeth.

Figure 1(a) demonstrates the glycolysis and demineralization processes in the mouth and the resulting hazards that can cause dental caries and erosion. Food debris on teeth provides abundant energy for the glycolysis of cariogenic bacteria, leading to massive acid production and a rapid drop in local pH. In addition, acid intake and gastroesophageal reflux can also lower the oral pH, resulting in the dissociation of Ca^2+^ and PO_4_^3-^ from hydroxyapatite in the teeth. Such mineral loss may cause pulp infection, tooth loss, and even systemic diseases. To quantify the pathological evolution, Ca^2+^ concentrations and pH values in saliva can be simultaneously measured by a flexible device that features a pair of electrochemical sensors with a 3-electrode configuration. The two working electrodes have been modified with Ca^2+^ ionophore II (ETH 129) and polyaniline (PANI) that can specifically detect Ca^2+^ and H^+^ levels as results of glycolysis and demineralization processes, while the shared reference electrode has been coated with polyvinyl butyral (PVB)/sodium chloride (NaCl) solid electrolyte to improve its long-term stability. The neutral carrier ETH 129 can form tremendously lipophilic 1 : 3 cation/ionophore complexes with free Ca^2+^, leading to superior sensitivity and selectivity towards Ca^2+^ sensing. Meanwhile, the protonation and deprotonation of the doped PANI film in acid and basic solutions can induce changes in membrane potential, thus enabling reversible pH monitoring. Detailed hierarchy structures in Figure 1(b) indicate a thin conduction layer made of gold and a poly(3,4-ethylenedioxythiophene) polystyrene sulfonate (PEDOT:PSS) layer underneath ETH 129 as an ion-to-electron transducer on a polyimide substrate sealed inside a biocompatible parylene package, resulting in a flexible sensor 5.80 × 4.95 × 0.03 mm^3^ in dimension. Due to low Ca^2+^ concentration [29] (~0.6 mM) and varying pH values from 6.2 to 7.6 in saliva [30], the sensing performance of the sensor has been improved by electrodepositing gold nanoparticles (AuNPs) on the electrode to increase the specific surface area (Figure S1). In addition, the modification of both PANI and PVB resulted in porous structures of the electrode, which can facilitate the enrichment of ions and improve the sensing sensitivity (insets in Figure 1(b)). The ultrathin configuration of the sensor allows either conformal contact on the surface of teeth or ready insertion into the diastema without discomfort (Figure S2). The silver-coated conductive magnet has a magnetic force of ~0.17 N, which is sufficient to carry the entire flexible system, enabling reliable electrical and mechanical interconnects between the sensor and other circuit modules (Figure S3). In addition, the reversible connections also facilitate the replacement of the modules when the sensor or battery is depleted without damaging the system.

Other system modules that include a wireless sensing circuit and a power supply circuit can all be fabricated using complementary metal-oxide-semiconductor (CMOS) fabrication techniques on thin polyimide substrates to achieve ultrathin formats. To adapt to the dimension of the tooth, which is typically 9 × 8 × 9 mm^3^ in dimension for a molar, both modules adopt a distributed design with all components situated on islands smaller than the surface area of teeth (Figure 1(c)). These islands are connected with bridges that overpass the diastema between teeth and allow bending to adapt to complex and curvilinear surfaces of teeth (Figure S4). The wireless sensing circuit not only measures the ambient temperature with an embedded temperature sensor but also measures the open-circuit potentials of the Ca^2+^ and pH sensors and filters high-frequency noise using high-impedance amplifiers (Figures 1(d) and S5). The signals are then read, processed, and transmitted wirelessly via Bluetooth. The wireless sensing circuit and the power supply circuit are assembled together with the sensor through conductive magnets, resulting in a complete system with an overall dimension of 31.5 × 8.5 × 1.35 mm^3^. The entire system was sealed in a waterproof insulator made of parylene C with a thickness of 1 *μ*m to ensure electrical stability in wet environments, except for the openings at the sensing electrodes. Moreover, customized medical dental braces with extreme flexibility were molded according to specific wearers (Figure S6), with prereserved spaces for integrating with the flexible systems, followed by encapsulating the systems using a biocompatible flexible silicone to further avoid battery leakage or circuit shorts and enhance the electrical stability under fluid conditions [31] (Figure 1(e)). The weight of the system and the entire electronic dental brace are 0.4 g and 2.8 g, respectively, which are equivalent to the weight of a candy (Figure S7). Benefiting from the flexibility of the system and the customizability of the brace, the electronic dental brace can adapt to the complex tooth topography and fit closely with tooth surfaces (Figures 1(f) and S8), leading to great mechanical stability for real-time oral health monitoring without disturbing the daily life of wearers.

### 2.2. Electrochemical Performance of the Ion-Selective Sensors

The electrochemical behaviors of the sensors were first studied. Three distinct sets of redox activities were observed during the electrochemical polymerization of PANI, as indicated by three pairs of anodic and cathodic current peaks, while the current intensity increased with the film thickness (Figure S9). The potential difference between the redox peaks increased with the scan rate, possibly due to an insufficient diffusion rate of anions leading to an enlarged polarization voltage (Figure S10). Besides, electrochemical impedance spectroscopy (EIS) shows a decrease of 83% in interfacial impedance at 1 kHz after modifying AuNPs, while the polymerization of PANI increased the electrode impedance compared to the Au electrode, indicating that the PANI film generated charge transfer hindrance between the electrolyte and the electrode (Figure S11). In addition, the use of the PVB/NaCl solid electrolyte provided the reference electrode with a saturated Cl^−^ concentration and, thus, a more stable potential that was hardly affected by ionic strengths in saliva, with an 87% improvement as compared with the bare Ag/AgCl electrode, resulting in further enhancement of the durability of the flexible system in complex saliva compositions (Figure S12).

**Figure 2:**
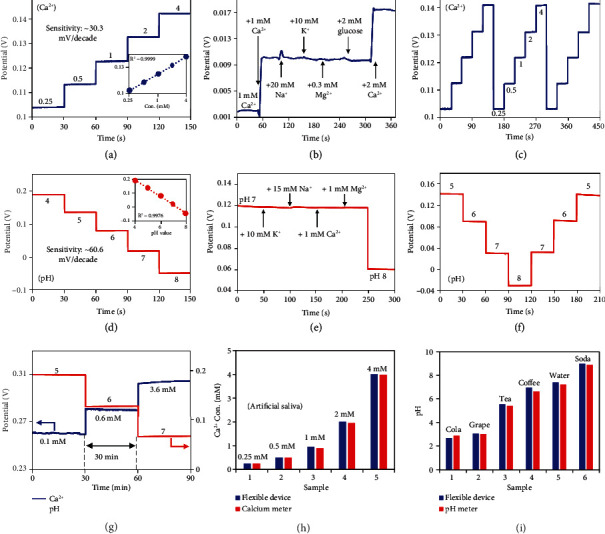
The sensing performance of ion-selective sensors. (a) The open-circuit potential response of a Ca^2+^ sensor with concentrations ranging from 0.25 to 4 mM. Inset: a linear fitting result of the Ca^2+^ sensing. (b) Selectivity and (c) reversibility of Ca^2+^ sensors. (d) The open-circuit potential response of a pH sensor with pH values ranging from 4 to 8. Inset: a linear fitting result of the pH sensing. (e) Selectivity and (f) reversibility of pH sensors. (g) Long-term stability of Ca^2+^ sensors and pH sensors with varying Ca^2+^ concentrations and pH values. The sensing performance of (h) Ca^2+^ sensors and (i) pH sensors in artificial saliva and daily beverages compared with a commercial calcium meter and a commercial pH meter, respectively.

The sensing capability of such ion-selective sensors was later characterized separately by potentiometric measurements in corresponding analyte solutions at a temperature of 37°C. The Ca^2+^ sensor exhibits a linear response at physiologically relevant concentrations from 0.25 to 4 mM with a sensitivity of 30.3 mV/decade, which approaches the Nernst limit of 30.77 mV/decade at 37°C [32] (Figure 2(a)). Besides, various electrolytes and metabolites (e.g., Na^+^, K^+^, Mg^2+^, and glucose) coexisting in saliva may influence the sensor accuracy. Negligible responses have been observed after adding interfering analytes into a 2 mM Ca^2+^ solution, suggesting outstanding selectivity of the sensor for discriminating and measuring target ions (Figure 2(b)). In addition, both unidirectional and bidirectional potentiometric measurements of the sensor show stable and fast responses to varying Ca^2+^ concentrations, with a 2.9% relative standard deviation (RSD) of sensitivity, indicating the excellent capability of the sensor for reversible, long-term measurements (Figures 2(c) and S13). Similarly, the pH sensor also exhibits a linear potential response to increasing pH values from 4 to 8 with a sensitivity of 60.6 mV/decade, providing favorable sensitivity and a linear range for salivary pH measurement (Figure 2(d)). The selectivity study shows a potential drift of around 2.4% as compared with the sensor sensitivity when adding related interferents (Figure 2(e)), and the reversible measurements only exhibit a small RSD of 0.7% (Figure 2(f)), indicating the capability of the sensor in adapting to the oral environment with complex interferents.

The stability of Ca^2+^ and pH sensors and characteristic variations between different sensors were also performed. Potential drifts of approximately 2.6 and 1.4 mV/h were observed for the Ca^2+^ sensor and pH sensor over a 90 min measurement period, while the average sensitivities were calculated to be 28.1 and 61.5 mV/decade, suggesting small sensor errors of less than 9.3% and 2.3% over an hour for the Ca^2+^ and pH sensors, respectively (Figure 2(g)). A potential deviation of only 1.1 mV/pH was observed by placing the Ca^2+^ sensor in different pH environments (Figure S14). Considering the relatively stable pH in the oral cavity, the Ca^2+^ sensing is robust and reliable in the saliva environment. In addition, six Ca^2+^ sensors and four pH sensors have been used to explore the variations among sensors. The Ca^2+^ sensors show sensitivities from 25.3 to 32.3 mV/decade with an RSD of 8.0%, while the pH sensors show sensitivities from 60.0 to 62.7 mV/decade with an RSD of 1.6% (Figure S15). The inconsistency in the initial potential that may result from manual modification processes can be resolved by performing a two-point calibration before actual measurements.

To further evaluate the sensor accuracy in the oral environment with complex compositions, the sensing performance of the Ca^2+^ sensor and pH sensor was measured in artificial saliva and beverages, respectively. Sensing results were found to be substantially consistent with commercial Ca^2+^ and pH meters (Figures 2(h) and 2(i)). The characterization results show high specificity and reproducibility of the system for Ca^2+^ and pH monitoring, indicating superb sensing performance that can adapt to the complex salivary composition and oral environment.

### 2.3. *In Vitro* Characterization of the Flexible System

**Figure 3:**
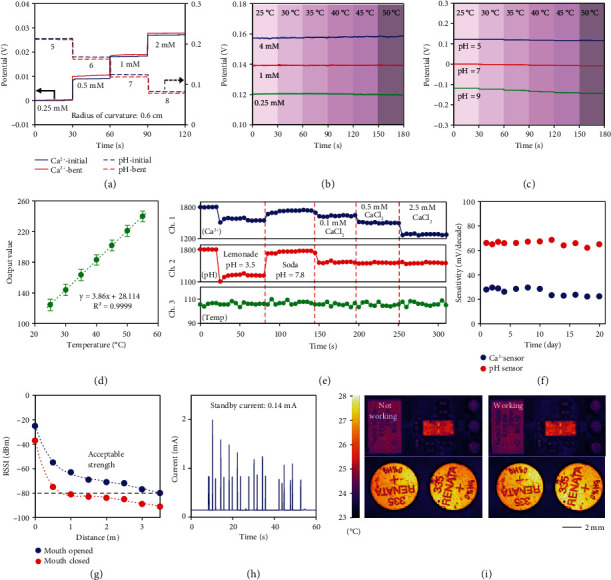
Mechanical, thermal, and long-term stability of the system. (a) Sensing results of a Ca^2+^ sensor and a pH sensor under bending with a curvature radius of 0.6 cm. Thermal stability of (b) a pH sensor and (c) a Ca^2+^ sensor upon different temperatures ranging from 25 to 50°C. (d) The linear fitting result of temperature sensing using embedded temperature sensors. *n* = 5; error bars indicate means ± SD. (e) Wireless, simultaneous monitoring of Ca^2+^, pH, and temperature *in vitro* under room temperature using the flexible system. (f) The lifespan of a Ca^2+^ sensor and a pH sensor. (g) Received signal strength measurements of the system under mouth opening and mouth closing conditions. (h) The power consumption of the system. (i) The temperature distribution of the circuit and batteries in nonworking and working modes.

The characterization of the entire system has been first conducted *in vitro* to study its physical properties as well as its long-term stability. When the system was bent at a curvature radius of 0.6 cm, no significant performance degradation was observed, with sensitivity deviations of 1.9% RSD and 0.4% RSD for the Ca^2+^ sensor and pH sensor, respectively (Figure 3(a)). Compared to the curvature radius of approximately 1.5 cm in the inner contour of the teeth, the system exhibits exceptional mechanical stability against external stress. The system has also been tested with repeated fluid flushing at a flow rate of 120 mL/min, which is much greater than the maximum salivary flow rate of ~5 mL/min [33]. No abrupt changes in potential were observed, only some high-frequency noises due to liquid fluctuations, suggesting the absence of material shedding due to the robust adhesion between the modifications and the electrodes (Figure S16). Besides, the thermal stability of the system was tested in a range of 25 to 50°C in line with the oral temperature [34]. Both the Ca^2+^ sensor and pH sensor maintained their sensing performance at different temperatures with slight potential drifts of 0.05 and 0.32 mV/°C, respectively (Figures 3(b) and 3(c)). The potential drift of the sensor can be further compensated by monitoring the real-time oral temperature using an embedded temperature sensor, which exhibits highly linear results with ambient temperature (Figure 3(d)). In addition, no corrosion or leakage was observed on the battery surface after continuous monitoring in artificial saliva for 24 h, indicating the excellent waterproof performance of the system package (Figure S17). Simultaneous system-level measurements of Ca^2+^, pH, and temperature in different aqueous solutions show excellent selectivity upon varying analytes, indicating high stability and accuracy of the system without mutual interference and short circuits (Figure 3(e)). Due to the elimination of bioactive components, the system can work continuously for at least 20 days with sensitivity deviations of 11.0% RSD and 2.5% RSD for the Ca^2+^ sensor and pH sensor (Figure 3(f)), respectively, suggesting promising potentials in long-term continuous oral health monitoring.

To demonstrate the portability of the system for daily wear and real-time measurements, received signal strength indication (RSSI) as a function of distance was measured under mouth opening and closing states, as signal strength can be greatly attenuated by the closed and humid oral cavity [35]. The transmission distance, which reached more than 3 m when the mouth was open and ~1 m when the mouth was closed, should be sufficient for daily communication between the system and mobile phones (Figure 3(g)). In addition, due to the optimized working sequence, the power consumption of the system was as low as 0.4 mW, resulting in a working period of more than 3 days when powered by 2 coin cells with a total capacity of 12 mAh (Figure 3(h)). For occasions where fast, real-time monitoring is not required, the circuit can also enter sleep mode and consume only ~55 *μ*A when not in operation, thereby providing longer battery life. The ultralow power consumption also ensures negligible heat generation by the circuit elements during operation, further enhancing the accuracy of temperature sensing. As shown in Figure 3(i), almost no heat was generated by the system during 3 h of operation, indicating favorable tissue compatibility with minimized thermal damage for long-term operation within the human body. The characterization results exhibit excellent mechanical and thermal properties as well as long-term stability of the system.

### 2.4. Ca^2+^ and pH Mappings on Extracted Teeth and Biocompatibility of the System

**Figure 4:**
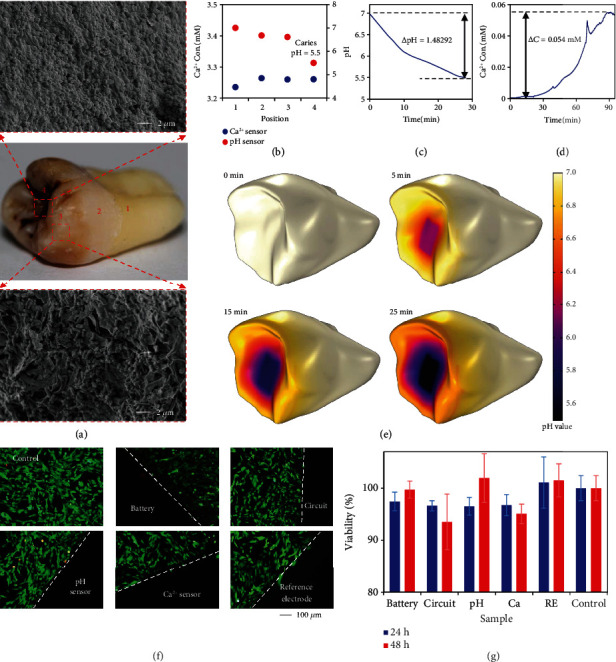
Ca^2+^ and pH mappings on extracted teeth and biocompatibility of the system. (a) Images of a tooth with active caries for Ca^2+^ and pH mappings. Insets: SEM images of the distribution of bacteria at a caries site (position 4) and a caries-affected site (position 3). (b) Ca^2+^ and pH measurements at different positions on tooth surfaces. (c) Dynamic changes in pH at the caries site (position 4) when the tooth was cultured in the artificial saliva. (d) The demineralization process of a tooth immersed in an acidic solution with a pH value of 4.3. (e) Simulation of bacterially produced lactic acid diffusing on tooth surfaces over time. (f) Fluorescent images obtained by Calcein-AM/PI double-stain assay showing the dyed viable (green) and dead (red) HGF-1 cells cocultured with batteries, circuits, Ca^2+^ sensors, pH sensors, and PVB/NaCl-coated reference electrodes. (g) Cell viability of HGF-1 cells obtained by CCK-8 assay after coculturing with different samples for 24 h and 48 h. *n* = 3; error bars indicate means ± SD.

The distributed configuration and flexible nature of the system enable multisite, continuous physiological monitoring, which was first characterized *in vitro* using extracted teeth. A tooth with active caries in pits and fissures has been used for Ca^2+^ and pH mappings. Scanning electron microscope (SEM) images show dense and thick spherical bacteria in the biofilm of the pits and fissures (marked as position 4), where the predominant cariogenic bacteria, Streptococcus mutans, are most prevalent (Figure 4(a)). Meanwhile, sparse rod-shaped bacteria (e.g., Lactobacillus) were observed on dental inclined planes and cusps (marked as position 3). Different species and densities of bacteria contributed to different levels of acid erosion, as indicated by the gradient discoloration on teeth [36]. Afterward, localized Ca^2+^ concentrations and pH values on tooth surfaces where the sensors were directly attached have been recorded in artificial saliva (Figure 4(b)). The signal obtained by the sensor can be considered as the synergistic result of local lesion release and fluid diffusion. The Ca^2+^ concentration on the dental cervix (marked as position 1) remained the same as the basal concentration of 3.23 mM in artificial saliva and rose to ~3.26 mM on the pits and fissures, cusps, and lingual surface (marked as position 2). In addition, the pits and fissures show the lowest pH of ~5.5, a critical pH at which demineralization occurs, while the pH values measured from the cusps and lingual surface were 6.61 and 6.78, respectively. These results offer Ca^2+^ and pH distributions that are highly consistent with discoloration profiles on carious tooth surfaces [37], providing a quantitative approach for estimating caries evolution.

To further reveal the evolution process of caries and their interaction with surrounding tooth surfaces, the dynamic acid production process was explored by continuous pH monitoring using a fresh carious tooth. The pH underwent a rapid drop from 7 to 6.1 during the first 10 min, then gradually decreased to 5.5, and leveled after 25 min, indicating the high viability of the cariogenic bacteria with a strong acid-producing capacity even in an *in vitro* environment (Figure 4(c)). The rapid drop in pH may be due to the large amounts of acid produced *in situ* by bacteria, while the slow drop can be attributed to acid production and diffusion, suggesting that dental caries may stem from various factors including bacterial activity and salivary flow rate. In addition, another tooth was immersed in a pH 4.3 solution to demonstrate the capability of dynamic Ca^2+^ monitoring during demineralization. The potential of the Ca^2+^ sensor remained stable until demineralization occurred and rose rapidly afterward (Figure S18). A total concentration change of 0.054 mM was observed within 90 min, suggesting a progressive, irreversible Ca^2+^ loss in acidic solutions that may lead to various oral diseases such as caries and dental erosion (Figure 4(d)).

The pH distribution on tooth surfaces was further studied by finite element analysis to reveal the dynamic acid generation and diffusion process. The pits and fissures of the tooth were set as active caries areas where abundant acid was produced, while the surroundings were set as saliva. The acid diffusion on tooth surfaces followed a transport of species in porous media since porous structured biofilms were observed in the SEM images. Detailed equations used to conduct simulation have been provided in the supplementary materials. The results show that the acid generated from the pits and fissures diffused rapidly to the surrounding within 5 min, leading to a pH reduction of ~0.2 at the inclined planes and cusps (Figure 4(e)). Moreover, a distinct distribution of pH that exhibited as gradually increasing pH values from the center to the periphery was observed after 25 min, indicating high consistency between the simulation results and experimental mapping results. The cavity without saliva flushing used in the measurement and simulation can mimic the oral environment of patients with xerostomia, a disease presumed to be highly associated with rampant caries [38]. The results show that in the absence of saliva flushing, rapid diffusion of bacteria-produced acid can lead to massive demineralization and enlarged caries lesions, thereby increasing the risk of rampant caries. The real-time sensing capability of the system enables monitoring of dynamic physiological parameters during the disease evolution, suggesting its potential applications in the pathogenesis analysis of rampant caries.

As the existence of the system does not disturb the function of bacteria on the teeth, it has also exhibited excellent biocompatibility with the presence of human gingival fibroblasts (HCG-1), which are major constituent cells around the integration locations of the flexible system. The cytotoxicity tests were conducted using Calcein-AM/Propidium Iodide (PI) double stain and Cell Counting Kit-8 (CCK-8) assays. Corresponding fluorescent images showing the cell viability of HGF-1 indicate the absence of cytotoxic effects during the coculture of the system and cells (Figure 4(f)). Notably, cells in contact with the devices (indicated by the dashed line) also maintained excellent viability, indicating the absence of separation or dissolution of encapsulating and modifying materials. Meanwhile, quantitative cytotoxicity obtained by CCK-8 tests shows that cells cocultured with various modules of the system maintained the viability of more than 90% (Figure 4(g)), which satisfies the required 70% cell viability for *in vivo* measurements as recommended by USP (ISO 10993-5) [39]. These results suggest that the waterproof packaging of parylene C and silicone prevents membrane-electrode separation and battery leakage in humid environments, indicating superior biocompatibility of the system for *in vivo* applications.

### 2.5. Real-Time, *In Vivo* Analysis of Oral Microenvironment Using the Flexible System

**Figure 5:**
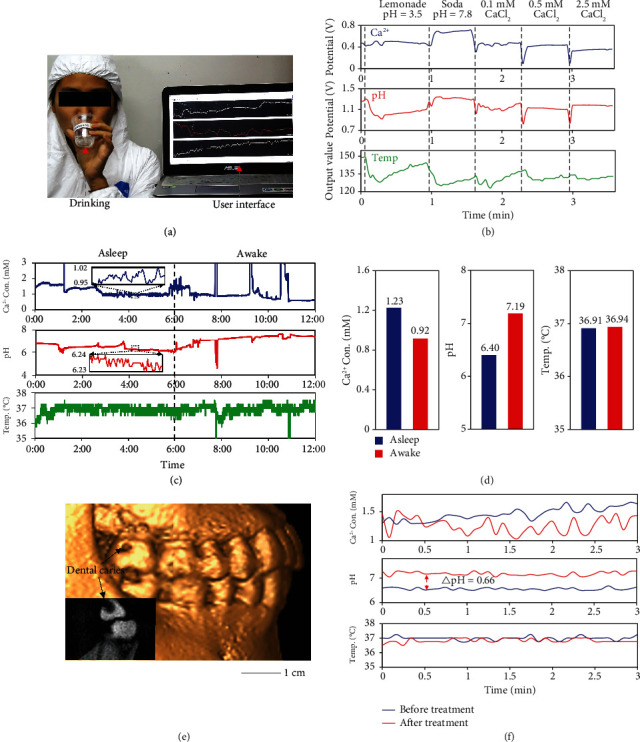
Real-time, *in vivo* analysis of oral microenvironment using the flexible system. (a) An image of the *in vivo* testing setup with a user interface and a human subject wearing a flexible system. (b) Simultaneous monitoring of Ca^2+^ concentration, pH value, and temperature when ingesting drinks and food additives with different Ca^2+^ concentrations and pH values. (c) Real-time, continuous monitoring of a healthy subject during asleep and awake periods. (d) Results of intraoral Ca^2+^ concentration, pH value, and temperature over 12 h. (e) A 3D reconstruction model of teeth based on CBCT images that shows the position of the caries cavity. (f) Analysis of salivary Ca^2+^, pH, and temperature of a patient before and after dental restoration treatment.

Prior to the *in vivo* integration of the system, sterilization processes through ultraviolet (UV) exposure and alcohol immersion may influence the functional coating on the sensors. Their influence on sensor signals has been characterized. The results indicate that the system maintains consistent sensitivity after disinfection processes with drifts in the initial potential for both sensors (Figure S19). The possible reason may be the changes in the composition of the functional coating caused by ozone and ethanol. The drifts in potential can be tackled through *in situ* calibration using food additives with known Ca^2+^ concentrations and pH values. Following the sterilization and the calibration, real-time physiological monitoring was then conducted on human subjects. Beverages and food additives with different Ca^2+^ concentrations and pH values were sequentially ingested by a subject (Movie S1), while the system continuously monitored the salivary parameters and transmitted the results to a computer (Figure 5(a)). The results show distinct curves corresponding to different Ca^2+^ and pH levels, with cyclical changes in temperature due to the temperature difference between the drinks and the oral environment (Figure 5(b)). Typically, the pH sensor shows stable and repeatable sensing results with a 7.9% RSD of sensitivity when drinking different beverages, indicating favorable reversibility during *in vivo* measurements (Figure S20). The erosive potential of different acidic drinks was also studied by measuring salivary pH at different time intervals after ingestion (Table S1). The results show that carbonated beverages took longer to neutralize the acid than fruit juices, while their pH values were comparable, indicating that the erosive potential is not only determined by the intrinsic pH of the beverage but also strongly influenced by its titratable acid content and its calcium-chelation properties that can effectively bind free calcium ions released from teeth.

The system has also been demonstrated to perform continuous oral health monitoring for a period of up to 12 h, which includes both asleep and awake periods (Figure 5(c)). Relatively stable Ca^2+^ concentration and temperature in saliva were observed during the measurement. Notably, the salivary pH exhibits a distinct circadian rhythm that is consistent with several previous studies [40, 41], with a slow drop to a minimum of 5.88 during sleep and a rapid rise to around 7.35 upon waking up, implying rhythmic homeostasis of intraoral physiological processes such as microbial activity, salivary secretion, and demineralization-remineralization dynamics. Moreover, a repeated 12-hour continuous monitoring test still showed similar trends (Figure S21). The results show that the sensor noise is less than 0.1 mM for Ca^2+^ and 0.01 for pH at the static state (insets in Figure 5(c)). However, similar large fluctuations were observed in all three curves, possibly due to relatively vigorous movements such as teeth grinding, saliva swallowing, and mouth breathing. Such motion artifacts may be further minimized by utilizing biocompatible adhesives capable of directly and reversibly fixing flexible systems on teeth or by eliminating the baseline drift and high-frequency noise through algorithms such as adaptive filtering. The protection of the brace and encapsulation materials ensures a good insulating and waterproof performance of the system without battery corrosion and leakage during continuous on-body measurements. However, behaviors such as chewing and grinding when eating solid food may wear down the encapsulation layer of the system, leading to acid corrosion, electrical shorts, and even battery leakage. Better biocompatibility may be achieved by introducing adaptively deformable encapsulations, such as cellular microstructures [42], microfluidic suspensions [43], and tissue-like materials [44, 45] to minimize package degradation. Thereafter, the mean values of the corresponding parameters during sleep and wakefulness were calculated (Figure 5(d)). The Ca^2+^ concentration at night was slightly higher than that during the day, while no significant difference was observed in salivary temperature. However, the mean pH during sleep time was as low as 6.40, which was appreciably different from the pH of 7.19 when awake. Decreased salivary pH at night may be caused by the reflux of esophageal acid [46] or due to a lower salivary flow rate as well as higher bacterial viability at night [41].

The system can also be used to evaluate treatment outcomes for patients who are conducting dental restoration treatment. Cone-beam computed tomography (CBCT) images show a distinct caries cavity at the cervical margin of a molar tooth, where the system was attached for parameter monitoring before and after the restoration treatment (Figure 5(e)). Localized pH values that were as low as 6.5 due to acid production by bacteria at the cavity have changed to around 7.2 after the treatment, resulting in reduced Ca^2+^ concentrations due to alleviative teeth decay (Figure 5(f)). At the same time, there was no obvious difference in temperature before and after treatment, indicating that physical restoration of teeth does not affect oral temperature.

### 2.6. Caries Risk Assessment Based on Clinical Conditions and Salivary Buffering Capacity

**Figure 6:**
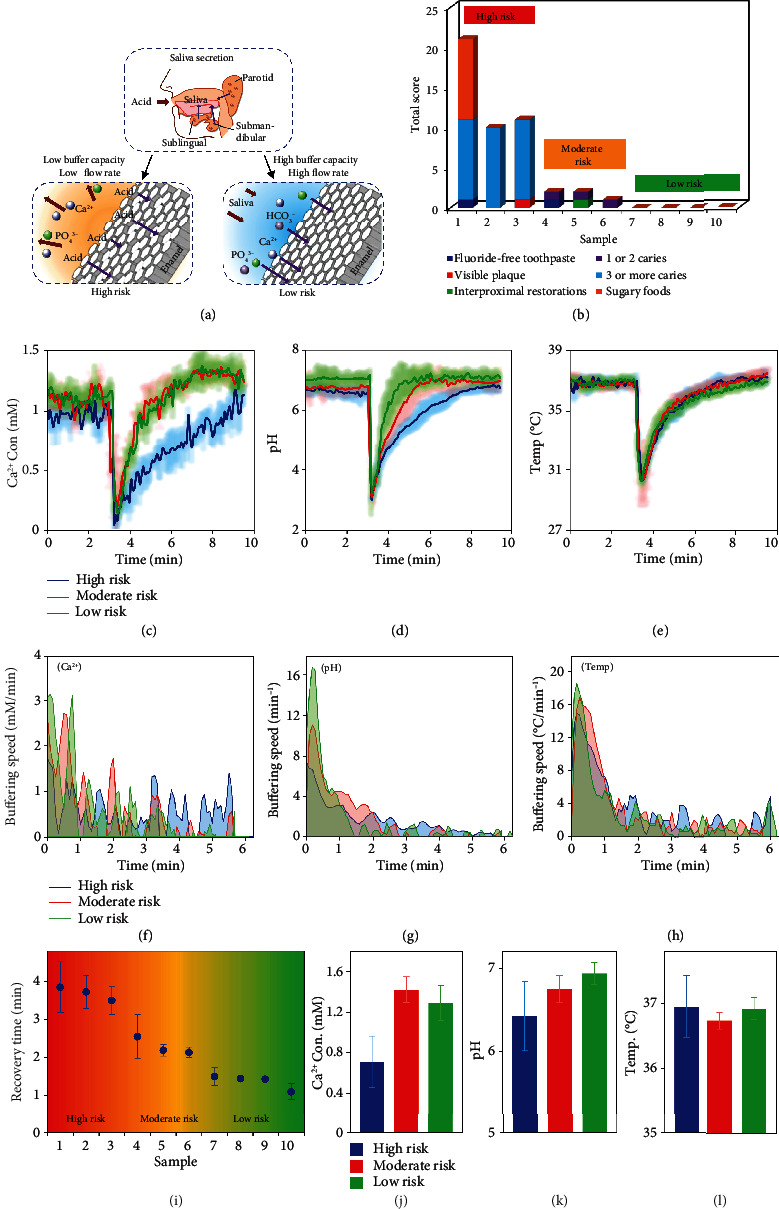
Caries risk assessment based on clinical conditions and salivary buffering capacity. (a) A schematic illustrating the relationship between salivary buffering capacity and dental caries. (b) Caries risk assessment for 10 subjects based on general health and clinical conditions. Representative sensing results of salivary (c) Ca^2+^ concentration, (d) pH value, and (e) temperature in different caries risk populations when applied to the same acid stimulation. *n* = 3; error bars indicate means ± SD. Corresponding buffering speeds of (f) Ca^2+^ concentration, (g) pH value, and (h) temperature in different caries risk populations. (i) Saliva recovery time after acid stimulation and risk classification of 10 human subjects. *n* = 3; error bars indicate means ± SD. Comparison of the resting (j) Ca^2+^ concentration, (k) pH value, and (l) temperature in different caries risk populations. *n* = 3; error bars indicate means ± SD.

Salivary buffering capacity and flow rate play an important role in oral health, as higher buffering capacity and faster flow rate facilitate the rinsing of food debris and the neutralization of acids, leading to a lower caries risk and a healthier dental status [47] (Figure 6(a)). However, the dynamic buffering process and the relationship between salivary parameters and oral diseases remain unclear. The continuous sensing capability of the flexible system enables its application in revealing such mechanisms by performing real-time measurements of salivary Ca^2+^ concentration, pH value, and temperature during exogenous stimulation. The oral environments of people with and without caries were treated as dynamic systems that respond to pulsed input in different manners due to the different saliva buffering capabilities in different persons. As the compositions of food are very complex and difficult to quantify, acidic beverages were selected as the pulsed input to stimulate oral environments. Subjects that are caries-free or suffering from dental caries were first classified as high-risk, moderate-risk, and low-risk subjects according to risk factors recommended by the American Dental Association [48] (Figure 6(b)). Generally, visible plaque and 3 or more caries were found in high-risk subjects, while no risk factor was found in low-risk subjects. Detailed information about the general health and clinical status of the subjects was summarized in Table S2. During the experiment, each subject was asked to drink 50 mL of lemonade, which caused the global changes in the concentrations of different chemicals, followed by the localized changes near the sensing site. Corresponding salivary parameters showing the saliva buffering process in different caries risk groups were recorded simultaneously throughout the test. Representative sensing results show that the moderate- and low-risk subjects exhibited a similar rapid drop in Ca^2+^ concentration after acid stimulation due to the strong calcium-chelation effect of citric acid [49], followed by a rapid rise within the first 2 min and a plateau afterward (Figure 6(c)). However, a more gradual recovery was observed in the high-risk subject who typically is believed to have slower saliva secretion in response to external stimulation. Besides, the pH recovery curve, which directly reflects the buffering capacity of saliva, was significantly different in all three subjects (Figure 6(d)). The low-risk subject returned to a normal pH within 2 min, while the high-risk subject took up to ~3.5 min, with the moderate-risk subject in between. Differences in recovery time can be attributed to different salivary properties, such as intrinsic pH, viscosity, composition, and flow rate, suggesting the dominant role of the buffering capacity in dental caries evolution. Similar conclusions have been proposed in previous studies [50–53] and have further been proven by continuous dynamic *in vivo* data obtained by the flexible system. In addition, no significant differences were found in the temperature profiles among the three subjects (Figure 6(e)). Afterward, the instantaneous speed of the saliva buffering process was also analyzed. For Ca^2+^ concentrations, low- and moderate-risk subjects experienced a buffering speed of 2 to 3 mM/min within the first minute and then gradually decayed to 0, while the high-risk subject maintained a low speed of less than 1 mM/min throughout the recovery process (Figure 6(f)). Slow buffering in high-risk subjects may be due to partial salivary gland dysfunction that can stem from nutritional deficiencies [54], medication usage [55], and psychosocial issues [56]. Besides, the pH buffering speed was clearly distinct in the three subjects. The instantaneous speed of pH change in the low-risk subject was up to 16.8 min^−1^ immediately after ingestion, well above the speed of 10.9 min^−1^ for the moderate risk and 6.4 min^−1^ for the high risk, indicating that people with a low caries risk have a higher buffering capacity and, thus, a faster neutralization process (Figure 6(g)). Additionally, the salivary temperature responded similarly to cold drinks in all three subjects, possibly due to a consistent heat transfer process in saliva (Figure 6(h)). These results demonstrate the dynamic buffering process of different caries risk groups, enabling more comprehensive caries risk assessment and etiological analysis in combination with conventional assessment models.

The saliva buffering processes of 10 subjects after acid stimulation were then used to quantify the caries risk (Figure 6(i)). The system shows highly consistent results with clinical assessment, with a recovery time of nearly 4 min for high risk, 2 to 3 min for moderate risk, and less than 2 min for low risk, indicating an integral role of the salivary buffering capacity in the pathogenesis and progression of caries. However, the Ca^2+^ concentration of several caries subjects did not return to its resting value, which refers to the salivary value in the absence of exogenous or pharmacological stimuli (Figure S22), while the pH of some healthy subjects after acid stimulation was slightly higher than the resting value (Figure S23), suggesting that the diverse components and complex physiological processes in saliva may lead to unpredictable individual differences that require more experimental and quantitative studies. Moreover, the relationship between resting oral parameters and caries risk has also been investigated. Resting Ca^2+^ concentrations were lower in high-risk subjects than in moderate- and low-risk subjects, consistent with several previous studies [57–59]. Calcium-rich saliva allows remineralization in the early stage of caries, while calcium-deficient saliva leads to a decrease in enamel crystallinity and, thus, an increase in the risk of dental caries, suggesting the important role of salivary Ca^2+^ in the dental pathogenesis [60] (Figure 6(j)). For subjects with high to low caries risk, a gradual increase from 6.42 to 6.94 has been observed in the mean resting pH, which may serve as an indicator for clinically diagnosing caries risk (Figure 6(k)). In addition, no significant differences were observed in the resting temperature of the subjects (Figure 6(l)). The capability of the flexible system for real-time, *in vivo* monitoring provides a novel approach for exploring the dynamic intraoral biological processes, suggesting potential applications in healthcare guidance, clinical diagnosis, and rehabilitation therapy. In addition, the portability and personalization of the miniaturized system allow for point-of-care testing at home, thereby saving medical resources and reducing economic costs.

## 3. Discussion

This paper presents a modularized flexible hybrid electronic system that can simultaneously monitor salivary Ca^2+^ concentration, pH level, and temperature for wireless real-time oral health monitoring. The distributed configuration of the system, which is 0.4 g in weight and 31.5 × 8.5 × 1.35 mm^3^ in dimension, has enabled conformal integration with curvilinear tooth surfaces. Characterization results indicate the excellent sensing properties of the system with high sensitivities of 30.3 and 60.6 mV/decade and slight potential drifts of 2.6 and 1.4 mV/h for Ca^2+^ and pH, respectively. Besides, the system exhibits superb mechanical and thermal stability against external stress and temperature fluctuations with a lifespan of over 20 days. Clinical applications of the flexible system have been demonstrated in Ca^2+^ and pH mappings on carious teeth, continuous physiological monitoring for more than 12 h, and assessing caries risk in 10 human subjects, suggesting its capability to offer quantitative data to assist clinic diagnosis and pathogenic mechanism study.

The flexible system can serve as a comprehensive platform by introducing different surface modifications to monitor diverse intraoral chemicals such as glucose, amylase, and tumor markers, as well as oral bacteria such as Streptococcus and Staphylococcus. In addition to sensing units, modulation components including micro-LEDs, stimulation electrodes, and drug-releasing carriers can also be integrated, enabling clinical applications in anti-inflammation, pain relief, and teeth whitening. To facilitate rapid deployment on teeth, biocompatible adhesives such as hydrogels, epoxies, and silicones that enable fast and reversible fixation of flexible systems on teeth with tuneable curing time and adhesive strength on the enamel may be developed. With the improved capability of large population deployment, the systems can be used to perform systematic clinical or daily measurements of larger populations. In combination with big data, artificial intelligence, and medical imaging, the dynamic data may facilitate the understanding of the interaction between oral physiological parameters and oral diseases, allowing sophisticated applications in pathological quantification, disease prevention, and personalized diagnosis.

## 4. Materials and Methods

### 4.1. Fabrication of Electrode Arrays

The fabrication process started with depositing 5 nm Cr/150 nm Au onto a polyimide film (25 *μ*m in thickness, DuPont de Nemours, Inc.), followed by patterning the electrode arrays via photolithography. The arrays were then coated with 1 *μ*m thick parylene C as an insulation layer, followed by etching with O_2_ plasma to define the sensing area. Subsequently, the Ag/AgCl reference electrodes were obtained by electroplating 500 nm Ag onto the Au electrodes, followed by immersing the resulting Ag electrodes into a 0.1 M FeCl_3_ solution for 90 s.

### 4.2. Preparation of Ca^2+^ Sensors and pH Sensors

All electrochemical modifications were performed using an electrochemical workstation (CHI760E, CH Instrument, Inc.). Au working electrodes were deposited with AuNPs by applying a constant potential of 0 V versus a commercial Ag/AgCl electrode for 30 s in a solution containing 50 mM HAuCl_4_ and 50 mM HCl. For Ca^2+^-selective electrodes, PEDOT:PSS was deposited onto the Au electrodes by galvanostatic polymerization at a current of 4 *μ*A for 740 s in a solution containing 0.01 M 3,4-ethylenedioxythiophene (EDOT, Aladdin Industrial Corp.) and 0.1 M poly(sodium 4-styrenesulfonate) (NaPSS, Sigma-Aldrich Corp.). A Ca^2+^-selective solution was obtained by dissolving 1 mg calcium ionophore II (ETH 129, Sigma-Aldrich Corp.), 0.55 mg sodium tetrakis [3,5-bis (trifluoromethyl)phenyl] borate (Na-TFPB, Sigma-Aldrich Corp.), 33 mg polyvinyl chloride (PVC, Sigma-Aldrich Corp.), and 64.45 mg bis(2-ethylhexyl) sebacate (DOS, Aladdin Industrial Corp.) in 660 *μ*L tetrahydrofuran (THF, Sigma-Aldrich Corp.). The resulting Ca^2+^-selective solution was then drop-casted onto the electrodes and dried overnight at room temperature. For H^+^-selective electrodes, PANI was electropolymerized by performing cyclic voltammetry from -0.2 to 1 V at a scan rate of 0.1 V/s for 25 cycles in a 0.1 M aniline (Shanghai Macklin Biochemical Co., Ltd.)/1 M HCl solution. For reference electrodes, 79.1 mg PVB (Butvar® B-98, Aladdin Industrial Corp.), 50 mg NaCl, 2 mg poly(ethylene glycol)-block-poly(propylene glycol)-block-poly(ethylene glycol) diacrylate (PEG-PPG-PEG, F127, Sigma-Aldrich Corp.), and 1 mg multiwall carbon nanotubes (Sigma-Aldrich Corp.) were dissolved into 1 mL methanol. Afterward, 1 *μ*L of the resulting solution was drop-casted onto the Ag/AgCl reference electrodes to minimize the potential drift.

### 4.3. Design and Fabrication of the Flexible Printed Circuit

The electronic components of the flexible circuit contained a low-dropout, micropower voltage reference (MAX6023EBT12+T, Maxim Integrated Products, Inc.), a quad micropower amplifier (AD8508ACBZ-REEL7, Analog Devices Inc.), a 12-bit analog-to-digital converter (ADC), and a Bluetooth low energy system on chip (BLE SoC) module (EYSNSNZWW, Taiyo Yuden Co., Ltd.) with an integrated high-performance antenna. Two coin-cell batteries (Renata 335, the Swatch Group Ltd.) were used to power the circuit. Moreover, Ag-coated conductive magnets (ShenZhen HongMing Magnetic Industry Co., Ltd.) with a thickness of 0.5 mm and a diameter of 1 mm were used as reversible electrical connections. Finally, the flexible circuit boards were encapsulated with 1 *μ*m thick parylene C to prevent leakage and improve biocompatibility.

### 4.4. Fabrication and Integration of Dental Braces

An impression body for each subject was first made using elastomeric impression material (Shandong Huge Dental Material Corp.). Customized dental models were then obtained by pouring fast-set plaster into the impressions and drying for 2 h. Afterward, another model with the same size as the flexible system was obtained by the above method, followed by bonding to the dental models using a single bond universal adhesive (3M Corp.). Dental braces were obtained by adapting polypropylene-based classic sheets (Sof-Tray™, Ultradent Products, Inc.) over the models and heating them in a vacuum former (Ultra-Form UP0250, Ultradent Products, Inc.). Flexible systems were encapsulated in the dental braces with biocompatible silicone (Ecoflex™ 00-30, Smooth-On, Inc.).

### 4.5. *In Vitro* Characterization of Sensors and Circuit

SEM images were obtained by a field emission gun scanning electron microscopy (Apreo 2, Thermo Fisher Scientific Inc.). Electrochemical impedance spectroscopy was performed in a 0.1 M KCl/5 mM [Fe(CN)_6_]^3-/4-^ solution over a frequency range from 0.01 Hz to 100 kHz with an amplitude of 5 mV. To characterize the sensing performance of the Ca^2+^ sensors, CaCl_2_ solutions with different Ca^2+^ concentrations were prepared and calibrated using a commercial Ca^2+^ meter (Pca-1-01, INESA Scientific Instrument Co., Ltd.). Selectivity study of the Ca^2+^ sensors was performed by subsequent addition of chlorides and glucose (1 mM Ca^2+^, 20 mM Na^+^, 10 mM K^+^, 0.3 mM Mg^2+^, and 2 mM glucose) into a 1 mM Ca^2+^ solution. For the pH sensors, McIlvaine buffer solutions with different pH values ranging from 3 to 9 were prepared and calibrated using a commercial pH meter (PHS-3C, INESA Scientific Instrument Co., Ltd.). Selectivity of the pH sensors was characterized by adding relevant interferences in physiological concentrations (10 mM K^+^, 15 mM Na^+^, 1 mM Ca^2+^, and 1 mM Mg^2+^) into a buffer solution with a pH value of 7. The sensitivity and linearity of the temperature sensor were obtained by immersing an encapsulated system in a constant temperature water bath (Shanghai Lichen-BX Instrument Technology Co., Ltd.) with a temperature range from 25 to 55°C. The power consumption of the circuit was measured by a source meter (Keithley 2400, Tektronix Inc.) with a constant supply voltage of 3.2 V. Thermal measurements were carried out using a thermal imaging camera (226s, FOTRIC Inc.).

### 4.6. Biocompatibility Tests

Cytotoxicity tests were conducted using the live/dead cell double staining kit and CCK-8 assay by exposing the samples to cultured HGF-1 cells (Procell Life Science & Technology Co., Ltd.). All the sensors, circuits, and batteries were sanitized with 75% alcohol and UV light, followed by bonding to the bottom of multiwell plates. HGF-1 cells were then cultured in the plates at 37°C under an atmosphere of 95% air and 5% CO_2_. For the live/dead cell double staining, cells were dyed by Calcein-AM (C1359, Sigma-Aldrich Corp.) and PI (C0080, Solarbio Science & Technology Co., Ltd.) after culturing for 24 h and 48 h, respectively. The fluorescent images were captured by a fluorescence microscope (BX51, Olympus, Corp.). For the CCK-8 assay, each plate was added with 10 *μ*L of CCK-8 solution (CK04, Dojindo Molecular Technologies, Inc.) after culturing the cells for 24 h and 48 h, respectively, followed by incubating for 2 h. The cytotoxicity was obtained by measuring the absorbance at 450 nm using a microplate photometer (Multiskan FC, Thermo Fisher Scientific, Inc.).

### 4.7. *In Vivo* Tests

*In vivo* measurements were approved by the Hospital of Stomatology at Tianjin Medical University with accreditation number TMUhMEC20210713. 10 subjects (3 males and 7 females), aged 22-27, without active periodontitis and oral mucosal disease, were recruited from the Tianjin University and Tianjin Medical University. All subjects gave informed consent before participating in the study. Clinical caries risk assessment was performed in compliance with the protocol recommended by the American Dental Association. Panoramic X-ray images were obtained by a digital panoramic imaging system (Orthophos XG, Dentsply Sirona Inc.). CBCT images were obtained by a cone beam 3D dental imaging system (Kavo 3D exam, Imaging Sciences International, Inc.). UV disinfection was performed by a UV/Ozone Cleaner (ProCleaner™, BioForce Nanosciences, Inc.).

## Data Availability

All data needed to support the conclusions in the paper are provided in the paper and the Supplementary Materials. The codes used in this study are available at https://github.com/imcort/BLE_PH_Monitor.

## Supplementary Materials

Supplementary Materials Figure S1: SEM images of a planar Au electrode and a AuNP-modified electrode. Figure S2: an image of a flexible sensor attached to teeth surfaces. Figure S3: reversible magnetoelectrical interfaces between different modules using conductive magnets. Figure S4: images of a sensor, a circuit, and two batteries connected in series under extreme bending. Figure S5: an illustration of the circuit design and detailed chip information for the intraoral system. Figure S6: a flexible customized dental brace under stretching, bending, and twisting. Figure S7: the weight of a flexible system and a dental brace embedded with a system. Figure S8: a subject wearing a dental brace integrated with a flexible system. Figure S9: CV curves of electrochemical polymerization of polyaniline with a scan rate of 0.1 V/s. Figure S10: CV curves of a PANI-modified electrode measured at different scan rates from 25 to 300 mV/s. Figure S11: EIS analysis of planar Au electrodes before and after different types of surface modifications. Figure S12: potential stability of a PVB-coated reference electrode and a solid-state Ag/AgCl electrode. Figure S13: reversibility of a Ca^2+^ sensor with reciprocating concentrations from 0.5 mM to 2 mM. Figure S14: potential stability of a Ca^2+^ sensor under different pH values. Figure S15: reproducibility of Ca^2+^ sensors (*n* = 6) and pH sensors (*n* = 4). Figure S16: stability of the system for Ca^2+^ and pH sensing with repeated fluid flushing at a flow rate of 120 mL/min. Figure S17: stability of system encapsulation in artificial saliva. Figure S18: the Ca^2+^ concentration measurement during the demineralization process when a tooth was immersed in an acidic solution (3 mL, pH = 4.3). Figure S19: the sensing performance of a Ca^2+^ sensor and a pH sensor disinfected by 75% alcohol immersion and ultraviolet exposure, respectively. Figure S20: *in vivo*, real-time pH monitoring when drinking different beverages with different acidities. Data were tested with the drink in the mouth for 30 s. Figure S21: continuous monitoring of intraoral physiology for 12 hours. Figure S22: representative sensing results of salivary Ca^2+^ concentration, pH value, and temperature in 3 dental caries patients after acid stimulation. Figure S23: representative sensing results of salivary Ca^2+^ concentration, pH value, and temperature in 3 caries-free people after acid stimulation. Table S1: intrinsic pH and measured salivary pH at different time intervals after consumption of different beverages. Table S2: caries risk assessment based on general health, clinical conditions, and salivary buffering capacity. Movie S1: wireless, real-time monitoring of salivary calcium, pH, and temperature.
