# Supplementary material for: Continuously Quantifying Oral Chemicals Based on Flexible Hybrid Electronics for Clinical Diagnosis and Pathogenetic Study
Source: Research (Wash D C). 2022 Aug 16;2022:9810129. doi: 10.34133/2022/9810129 (PMC9414179; doi:10.34133/2022/9810129)

Supplementary Text

**Simulation of acid diffusing on tooth surfaces**

Commercial finite-element analysis software Comsol was used to study the diffusion behaviors of acid in saliva. The 3D molar was modeled by Rhinoceros 3D software. The pits and fissures of the molar were set as the caries active zone with a H+ concentration growth rate of 2.04 × 10-9 M/s. The peripheral environment was set as saliva with a diffusion coefficient of 2.88 × 10-9 m2/s and a viscosity of 1.05 cP. The porosity was set to 0.5 for the pits and fissures, and 0.1 for the inclined planes to mimic a real tooth surface. The initial H+ concentration in saliva was 1 × 10-7 M, and the saliva temperature was 37℃. The acid diffusion on tooth surfaces follows the transport of species in a porous media model whose governing equations are as follows:

where is the porosity of the medium, is the concentration of H+ in the liquid, is the amount adsorbed to solid particles, is the velocity field describing the convection, is the dispersion tensor, is the effective diffusion, is a reaction rate expression that can account for reactions in the liquid, and is an arbitrary source term. The effective diffusion model was set to the Millington and quirk model with the following equations:

where is the fluid diffusion coefficient. Besides, the acid diffusion in saliva can be considered as a free diffusion model, whose equations are:

where is the diffusion coefficient.

Supplementary Figures and Tables


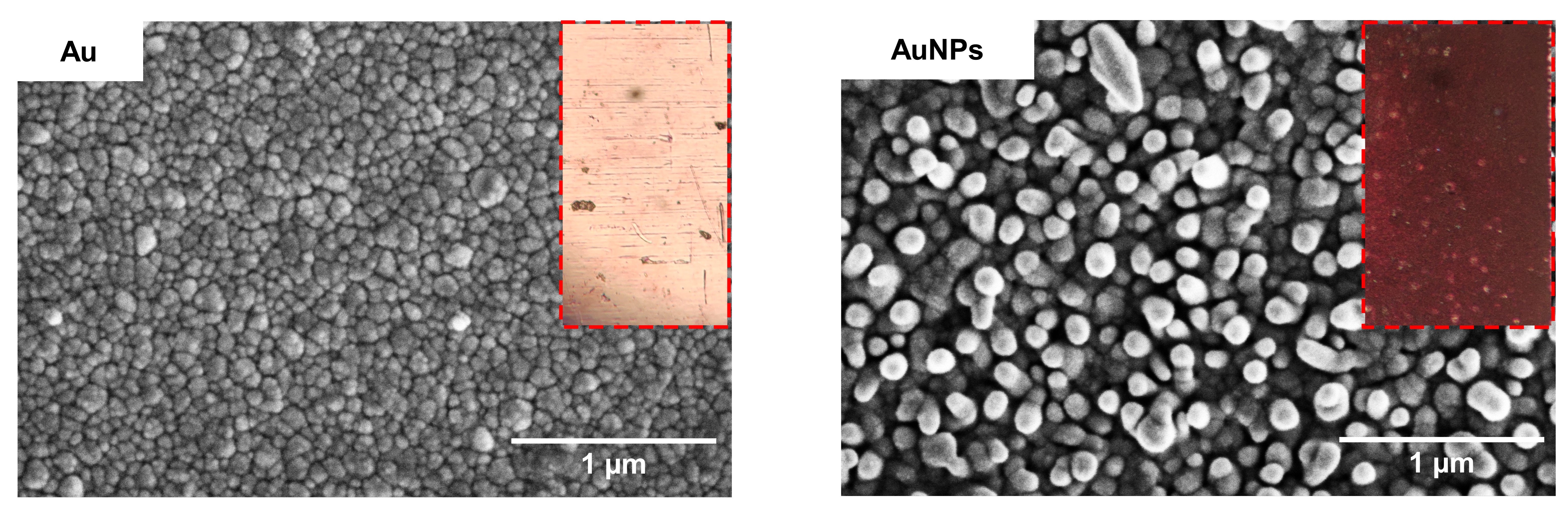


**Figure S1**. SEM images of a planar Au electrode and a AuNPs-modified electrode, respectively. Insets: corresponding optical images of the electrodes.


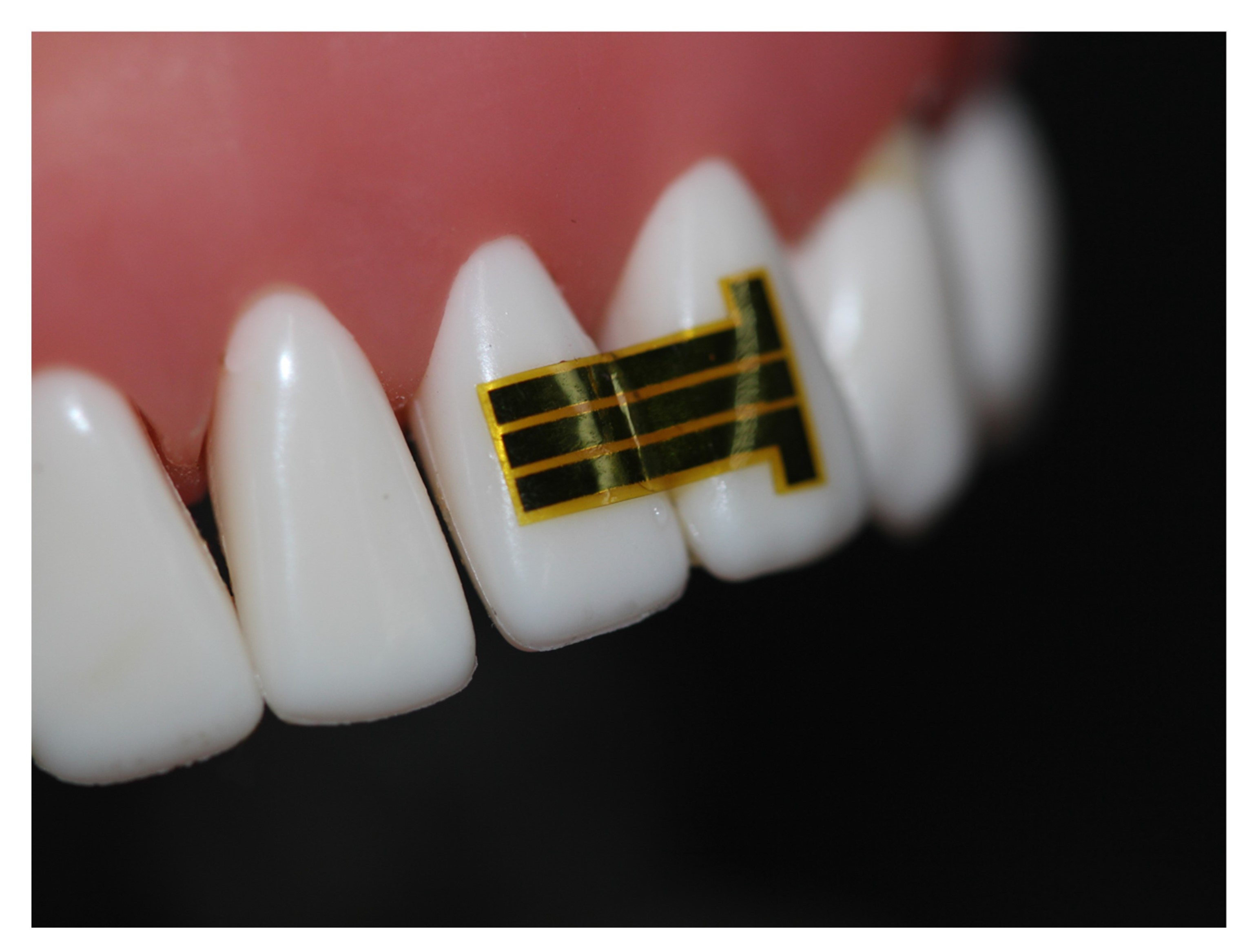


**Figure S2**. An image of a flexible sensor attached to teeth surfaces.


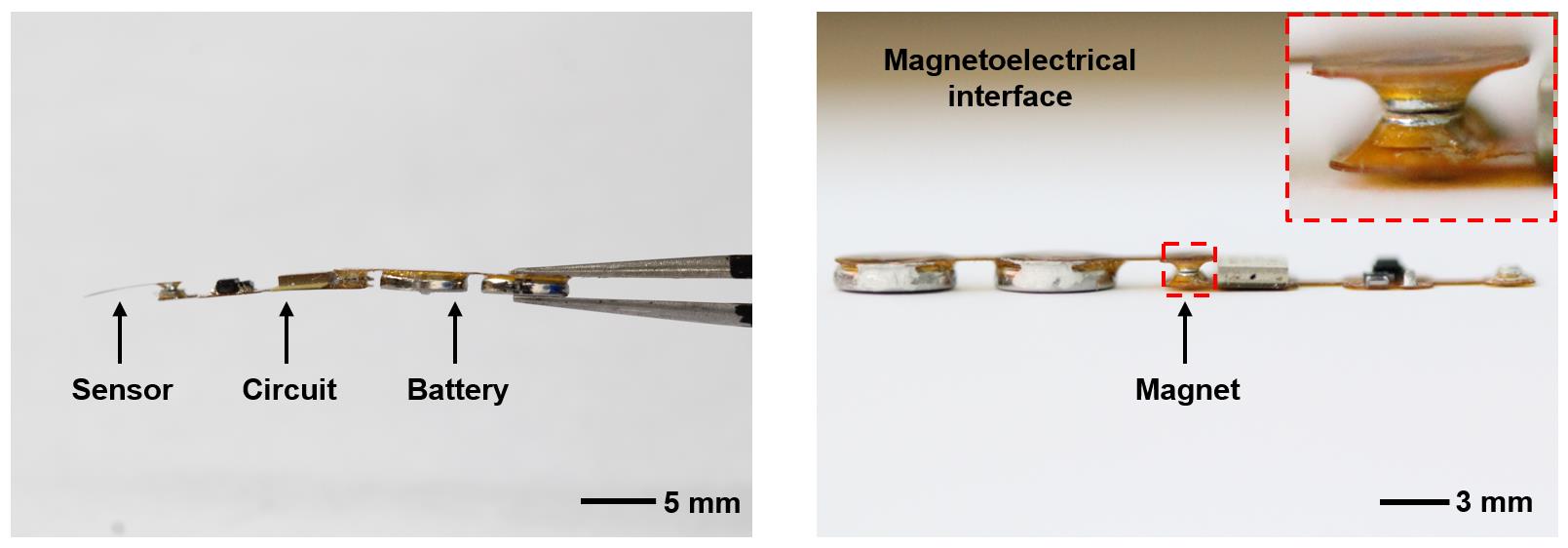


**Figure S3**. Reversible magnetoelectrical interfaces between different modules using conductive magnets.


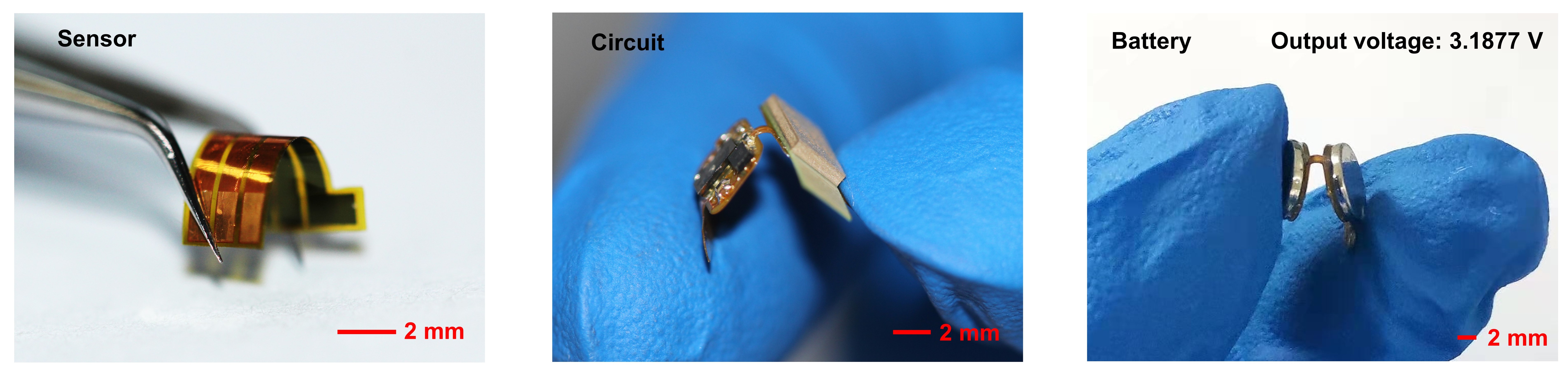


**Figure S4**. Images of a sensor, a circuit, and two batteries connected in series under extreme bending.


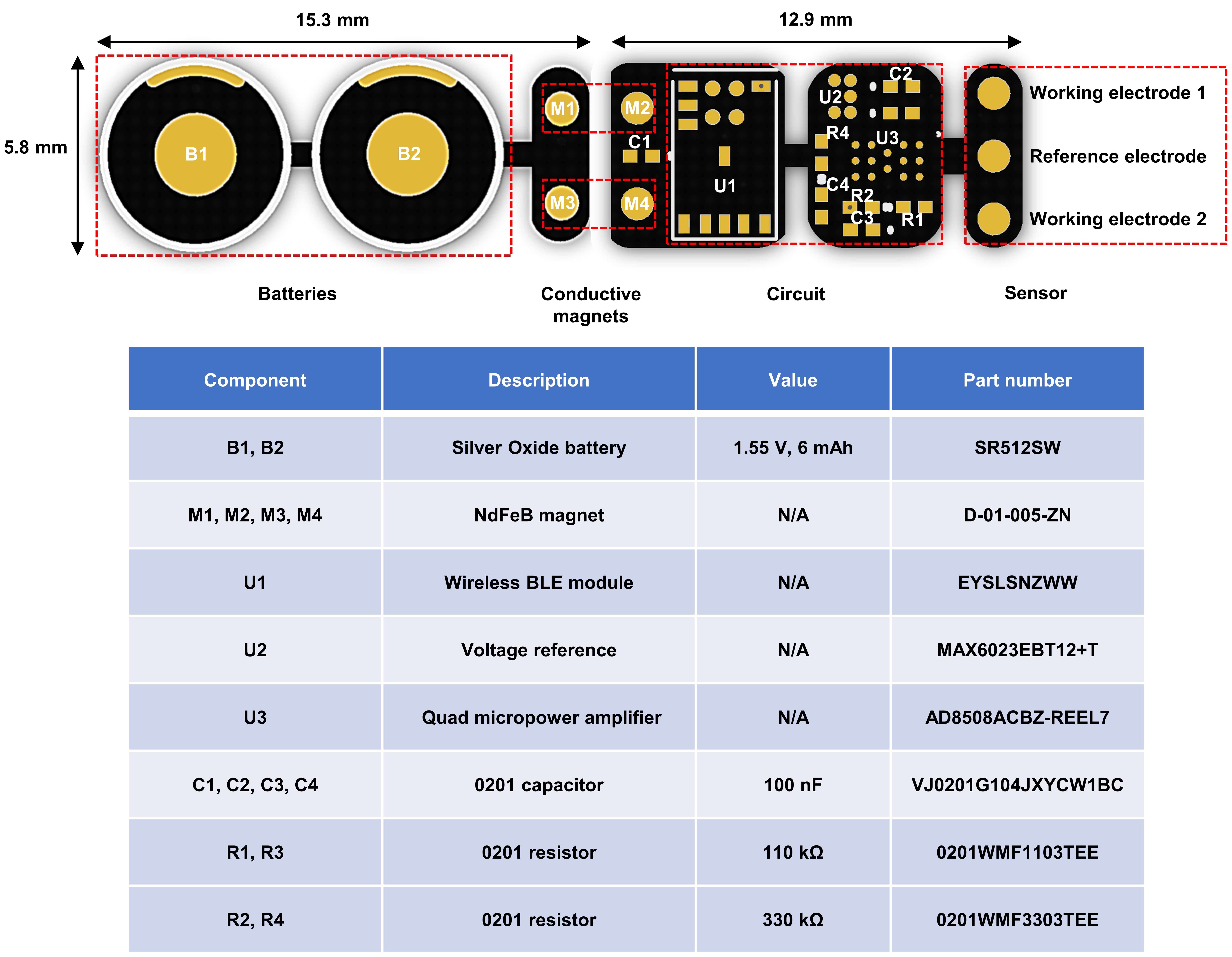


**Figure S5**. An illustration of the circuit design and detailed chip information for the intraoral system.


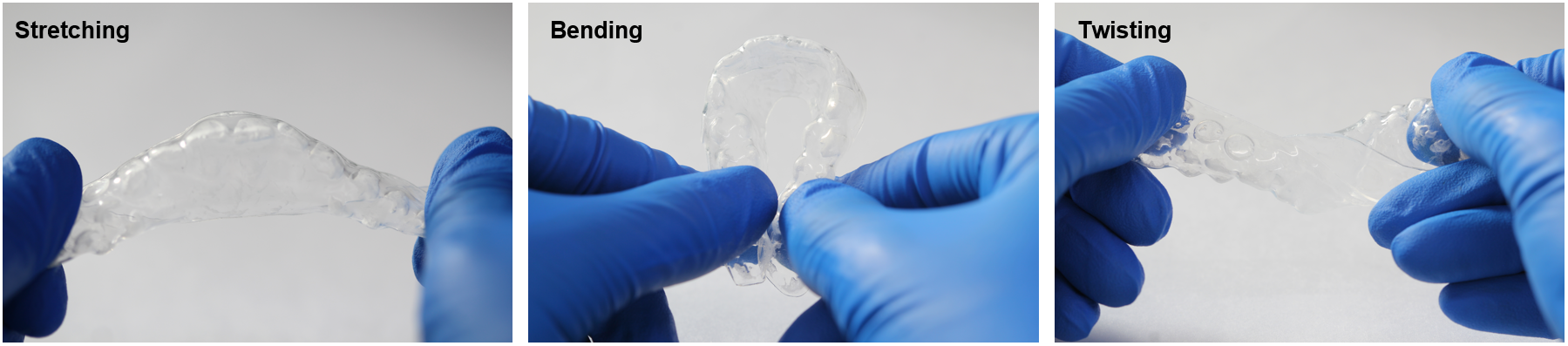


**Figure S6**. A flexible customized dental brace under stretching, bending, and twisting.


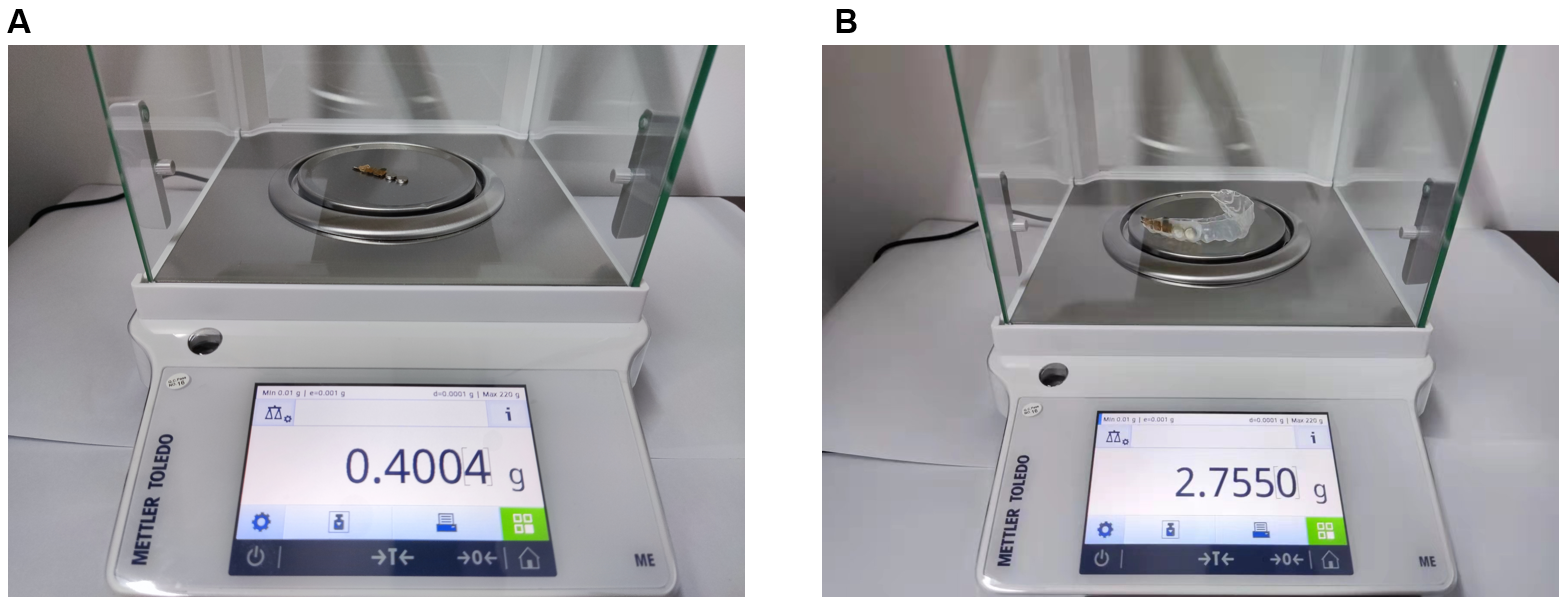


**Figure S7**. The weight of a flexible system and a dental brace embedded with a system, respectively.


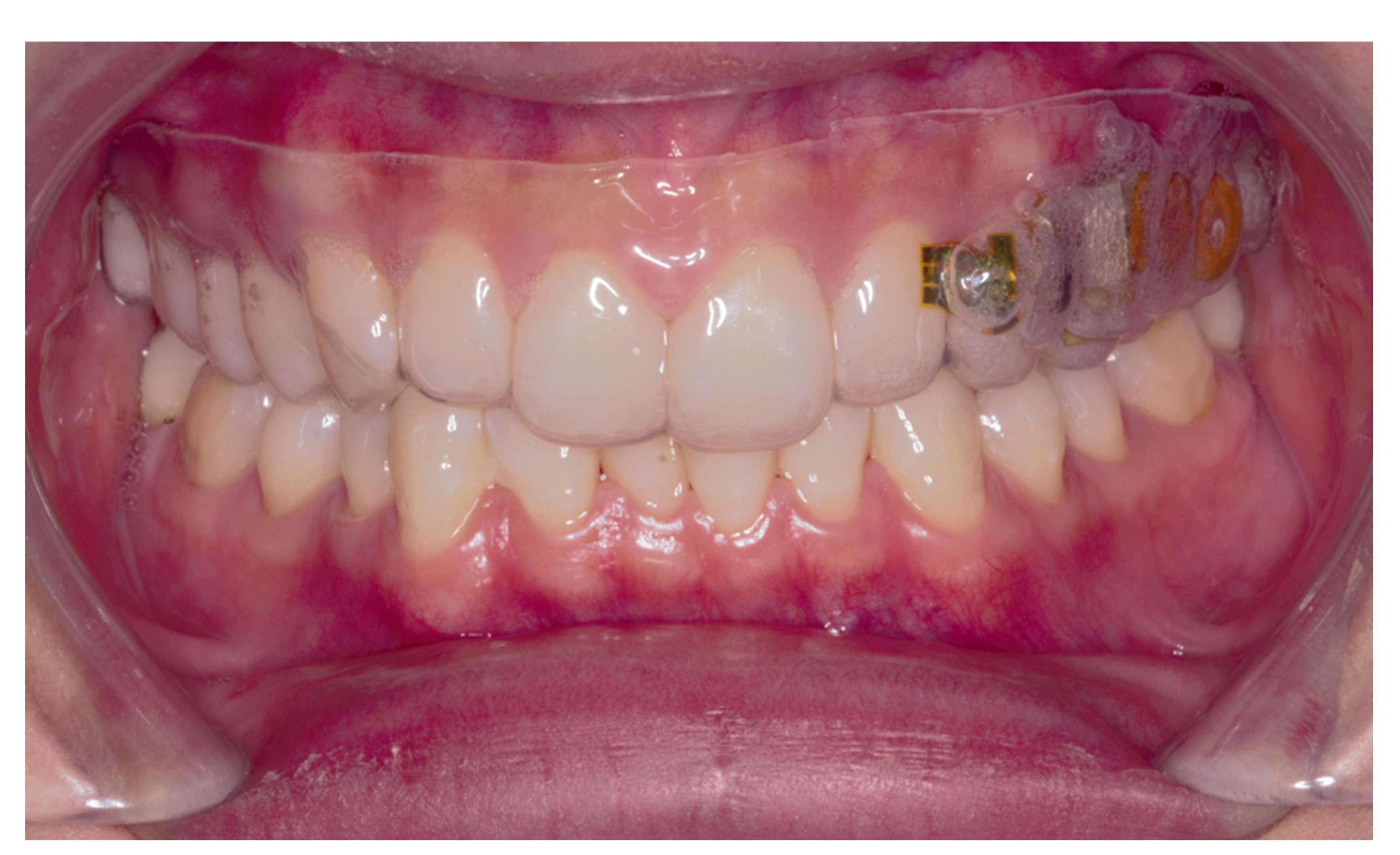


**Figure S8**. A subject wearing a dental brace integrated with a flexible system.


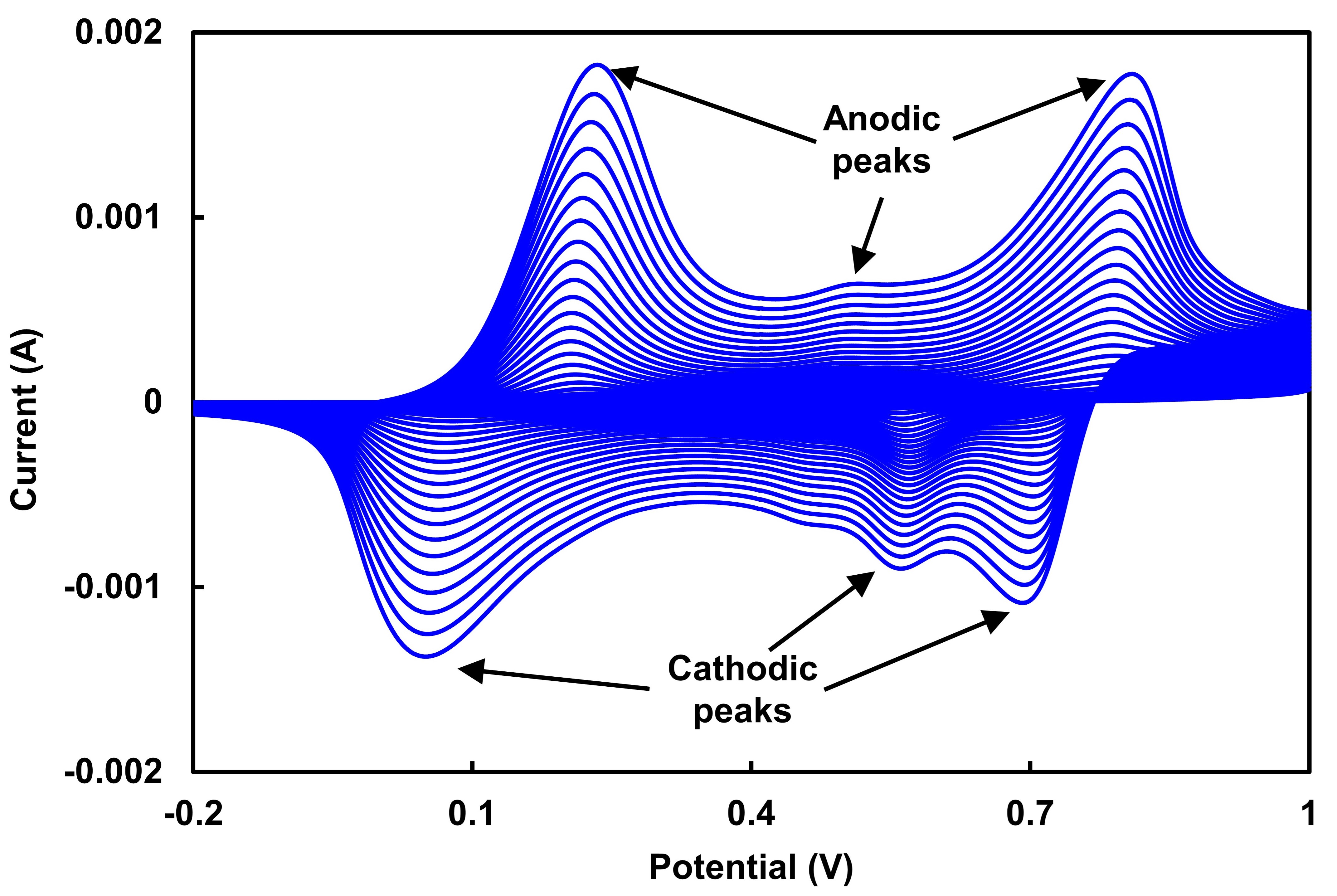


**Figure S9**. CV curves of electrochemical polymerization of polyaniline with a scan rate of 0.1 V/s.


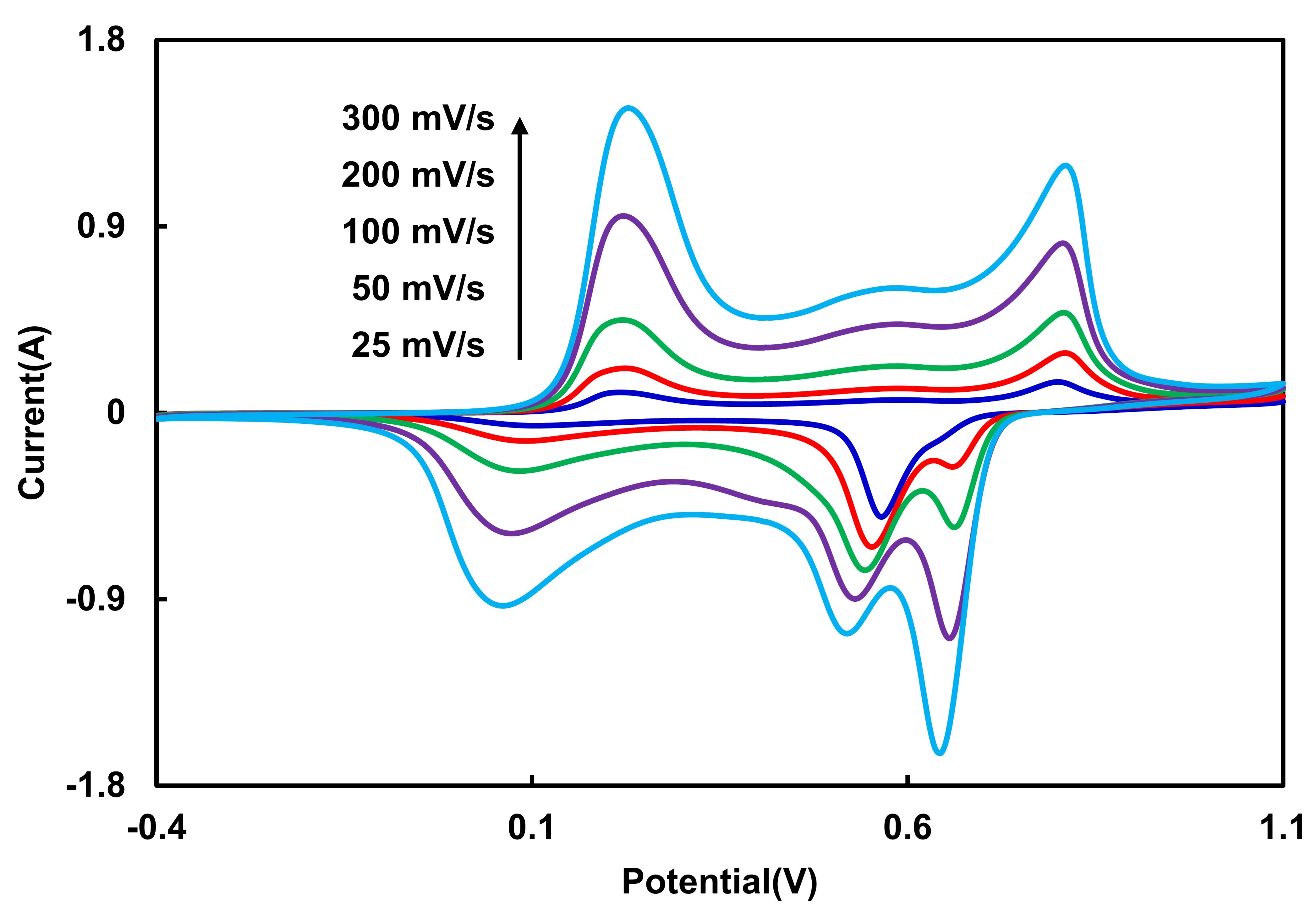


**Figure S10**. CV curves of a PANI-modified electrode measured at different scan rates from 25 to 300 mV/s.


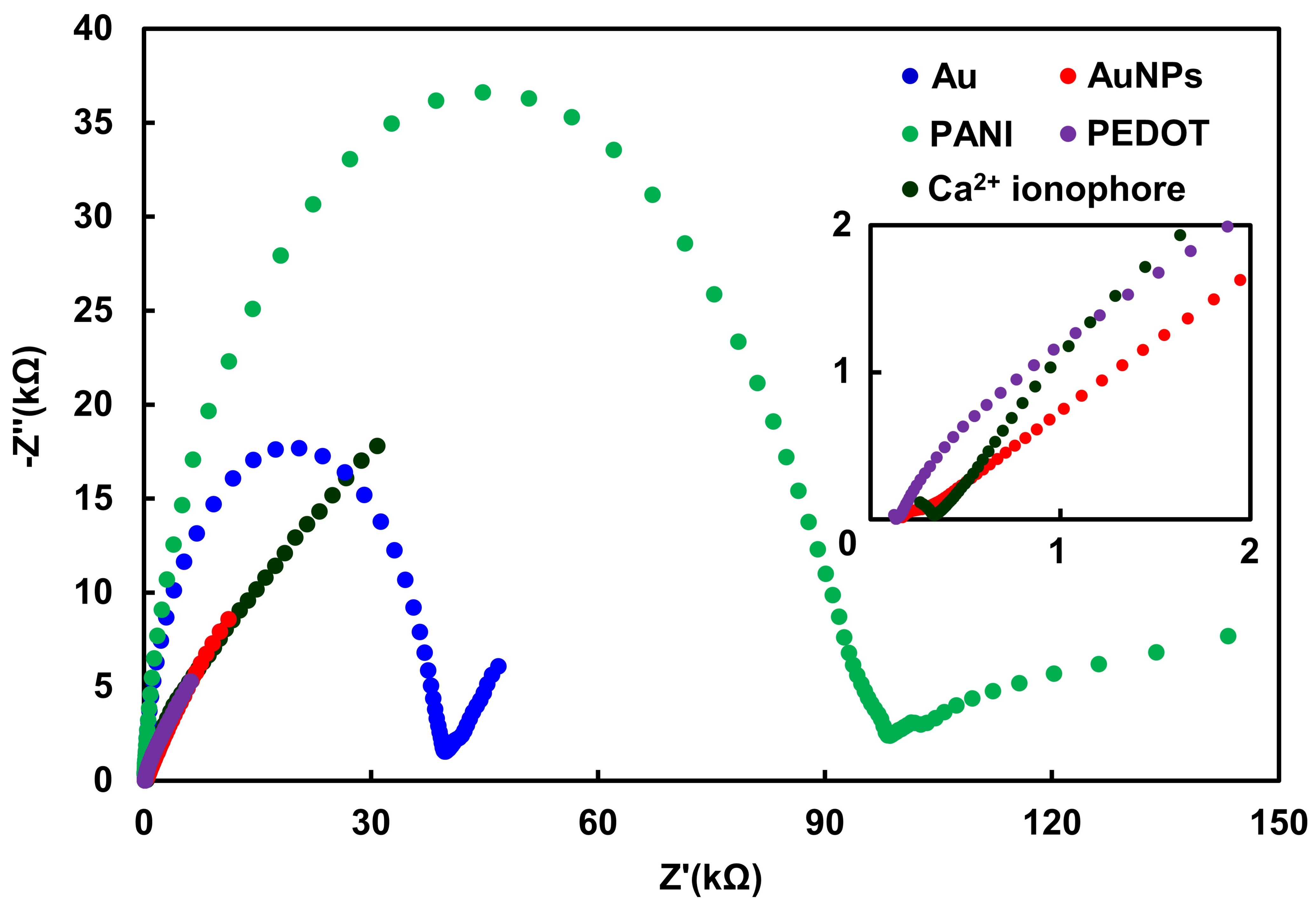


**Figure S11**. EIS analysis of planar Au electrodes before and after different types of surface modifications.


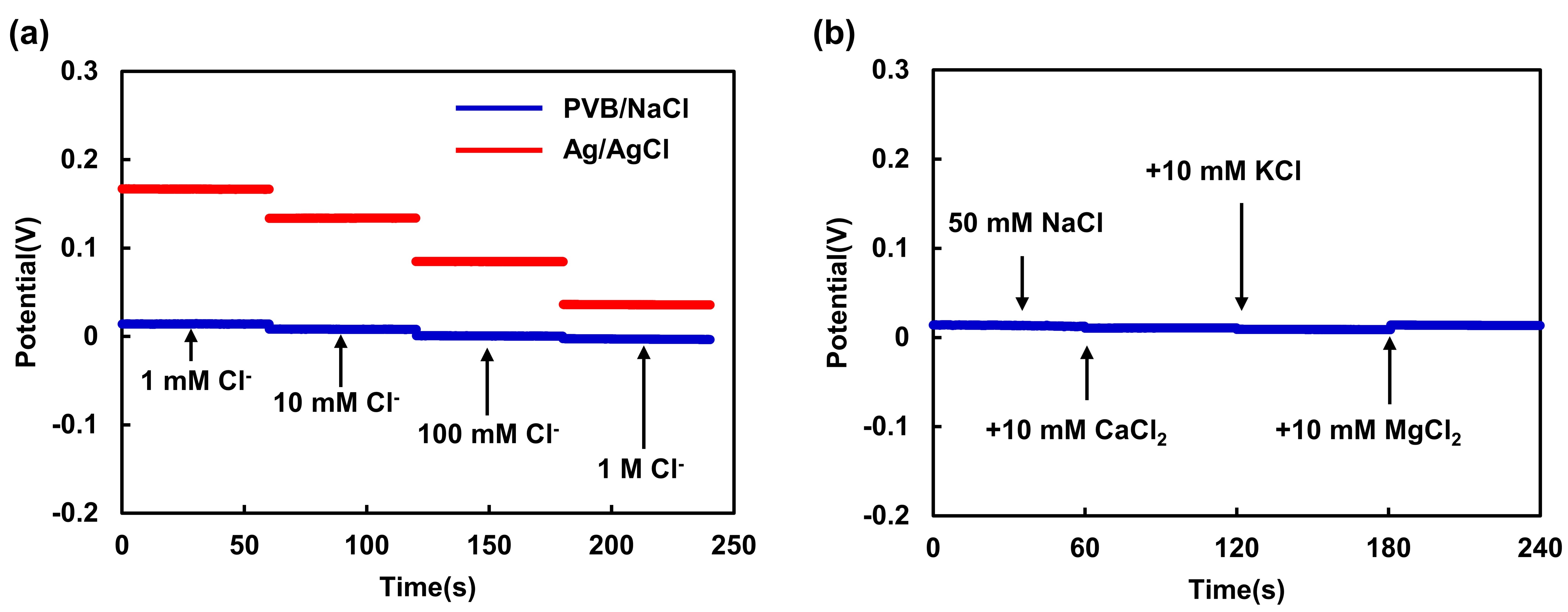


**Figure S12**. Potential stability of a PVB-coated reference electrode and a solid-state Ag/AgCl electrode (versus a commercial Ag/AgCl electrode) (a) under different Cl- concentrations and (b) in solutions containing 50 mM NaCl and 10 mM of different cationic solutions.


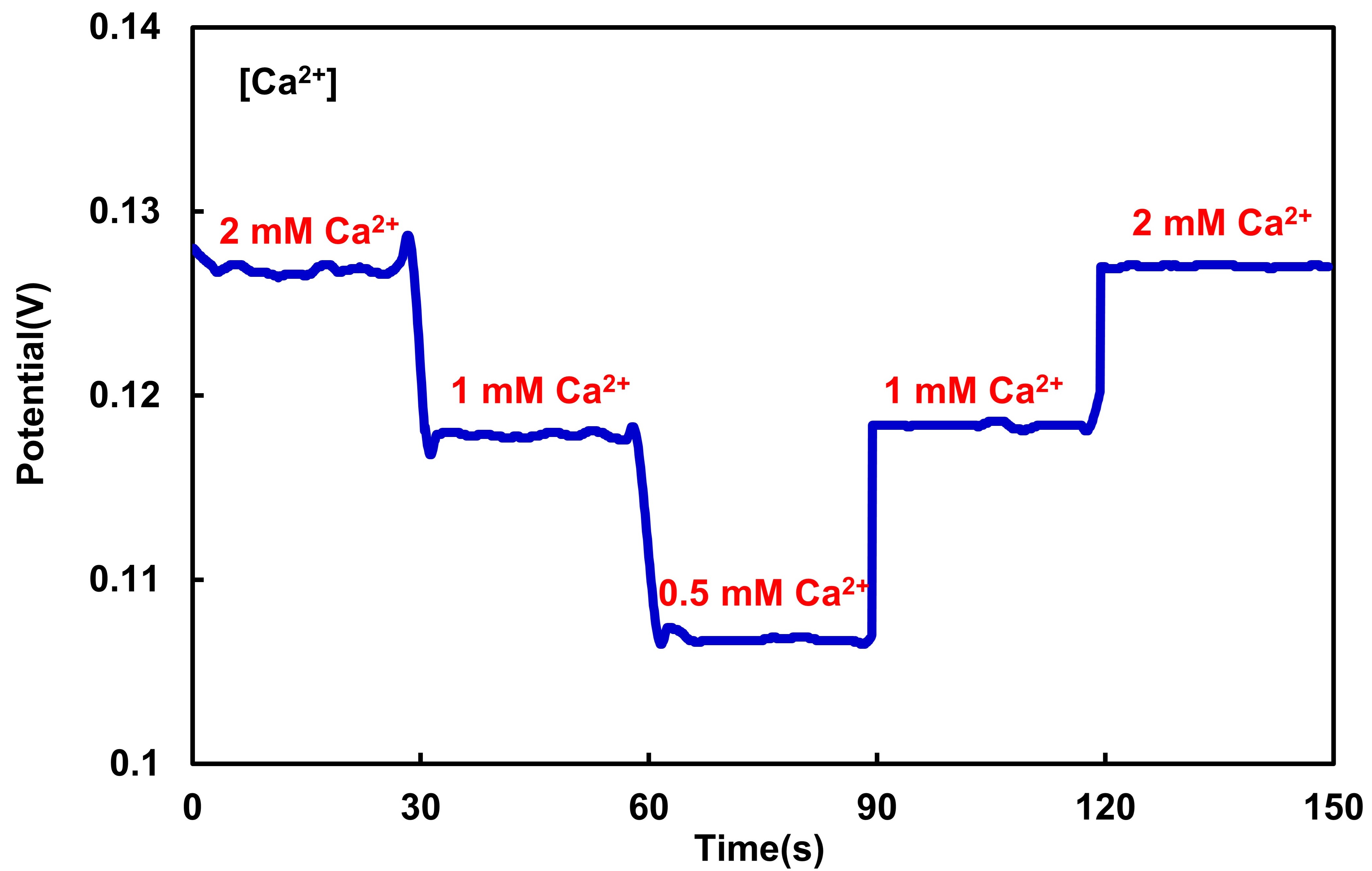


**Figure S13**. Reversibility of a Ca2+ sensor with reciprocating concentrations from 0.5 mM to 2 mM.


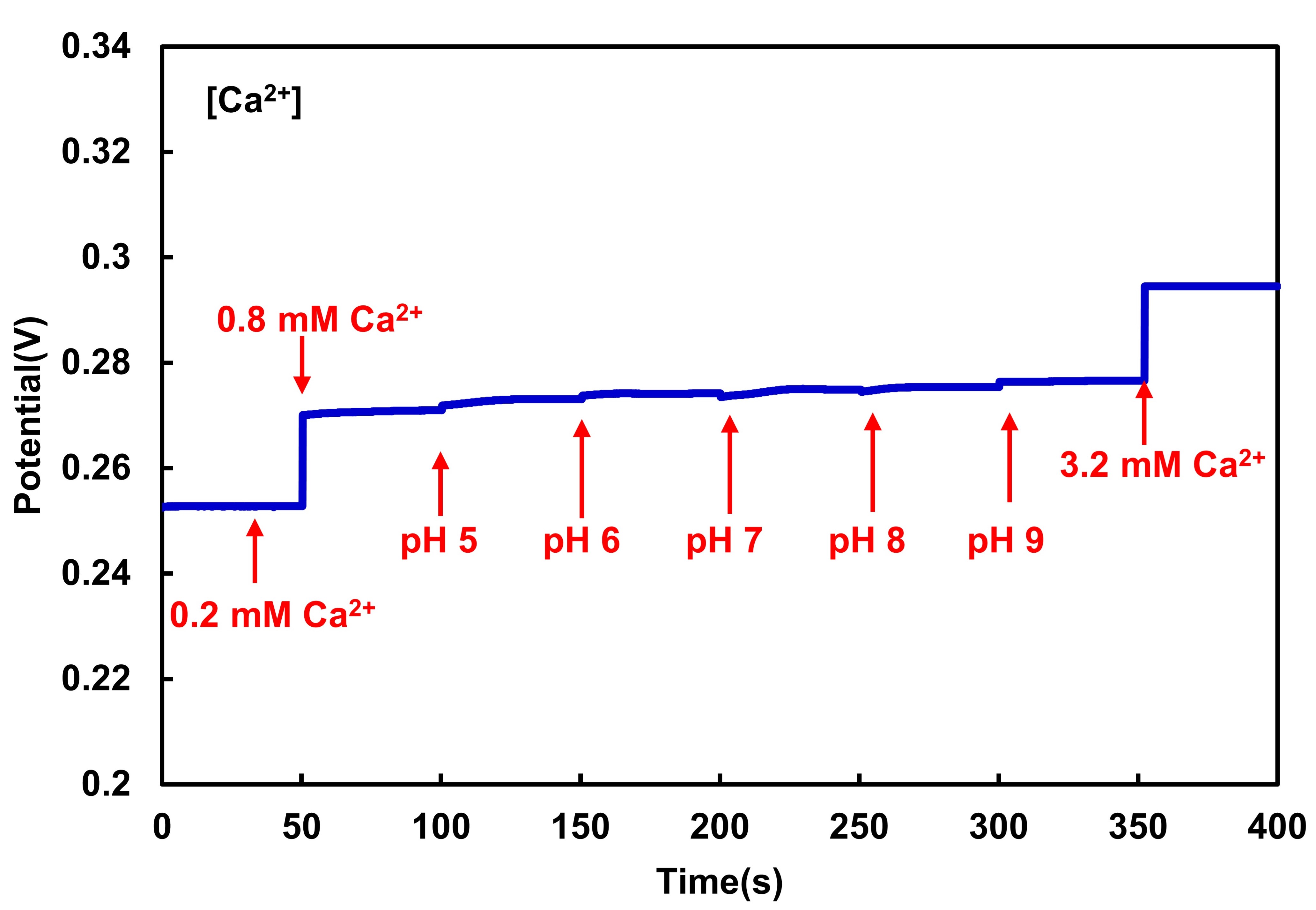


**Figure S14**. Potential stability of a Ca2+ sensor under different pH values.


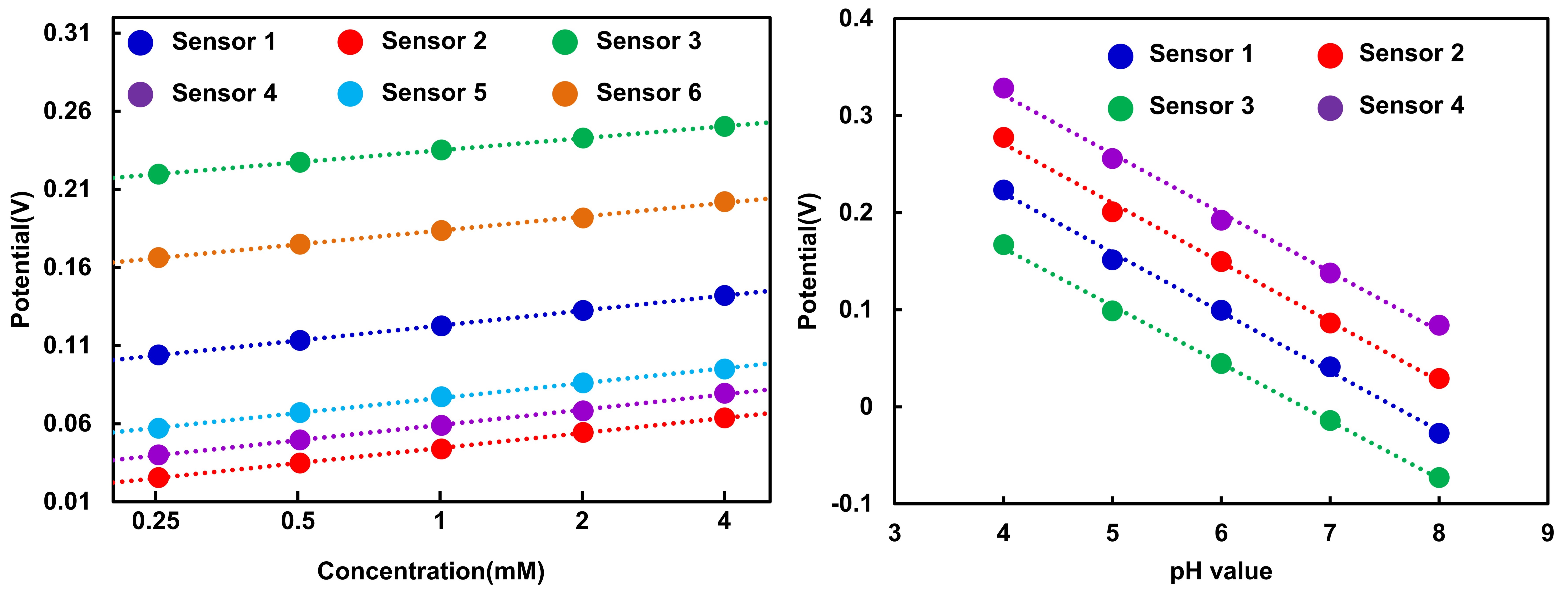


**Figure S15**. Reproducibility of Ca2+ sensors (*n* = 6) and pH sensors (*n* = 4).


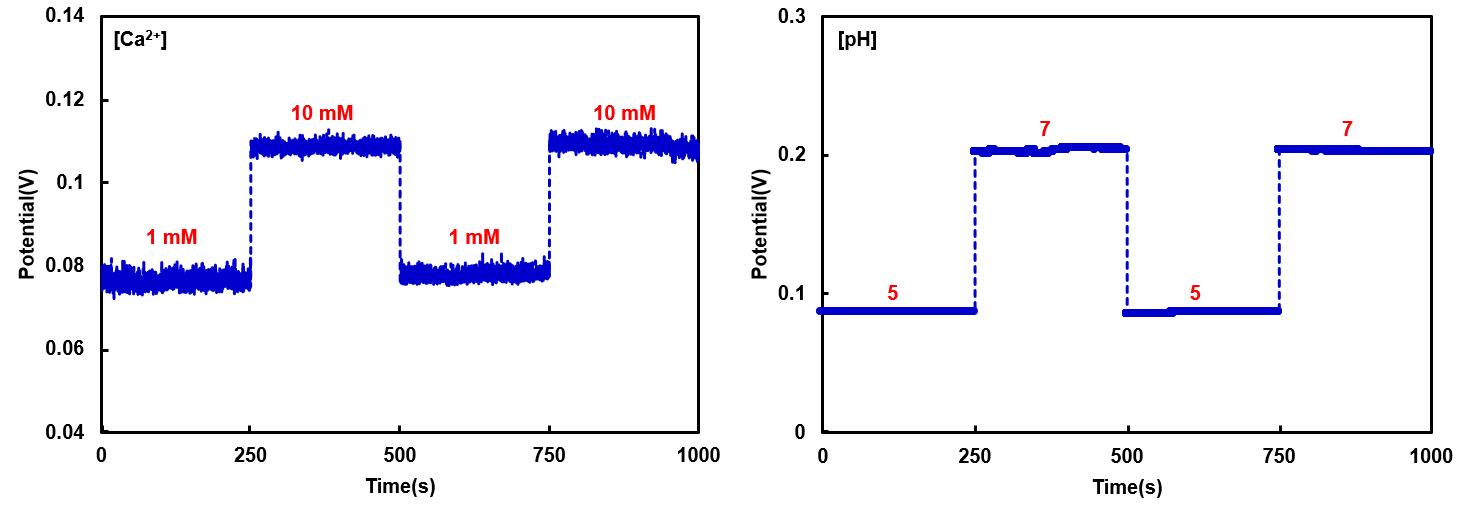


**Figure S16**. Stability of the system for Ca2+ and pH sensing with repeated fluid flushing at a flow rate of 120 mL/min.


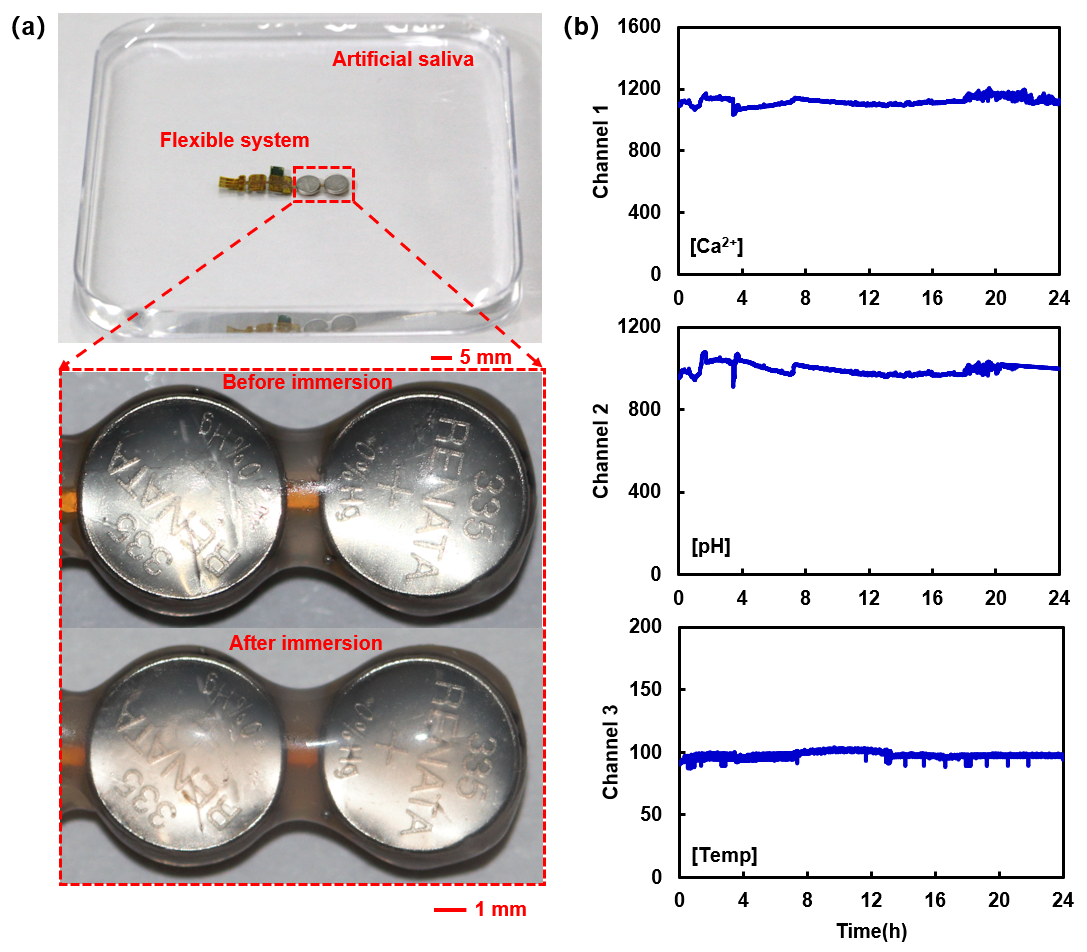


**Figure S17**. Waterproof capability of the system in artificial saliva. (a) Images of the system immersed in artificial saliva for 24 h. Inset: Encapsulated batteries before and after immersion. (b) Continuous measurement of Ca2+, pH, and temperature in artificial saliva for 24 h.


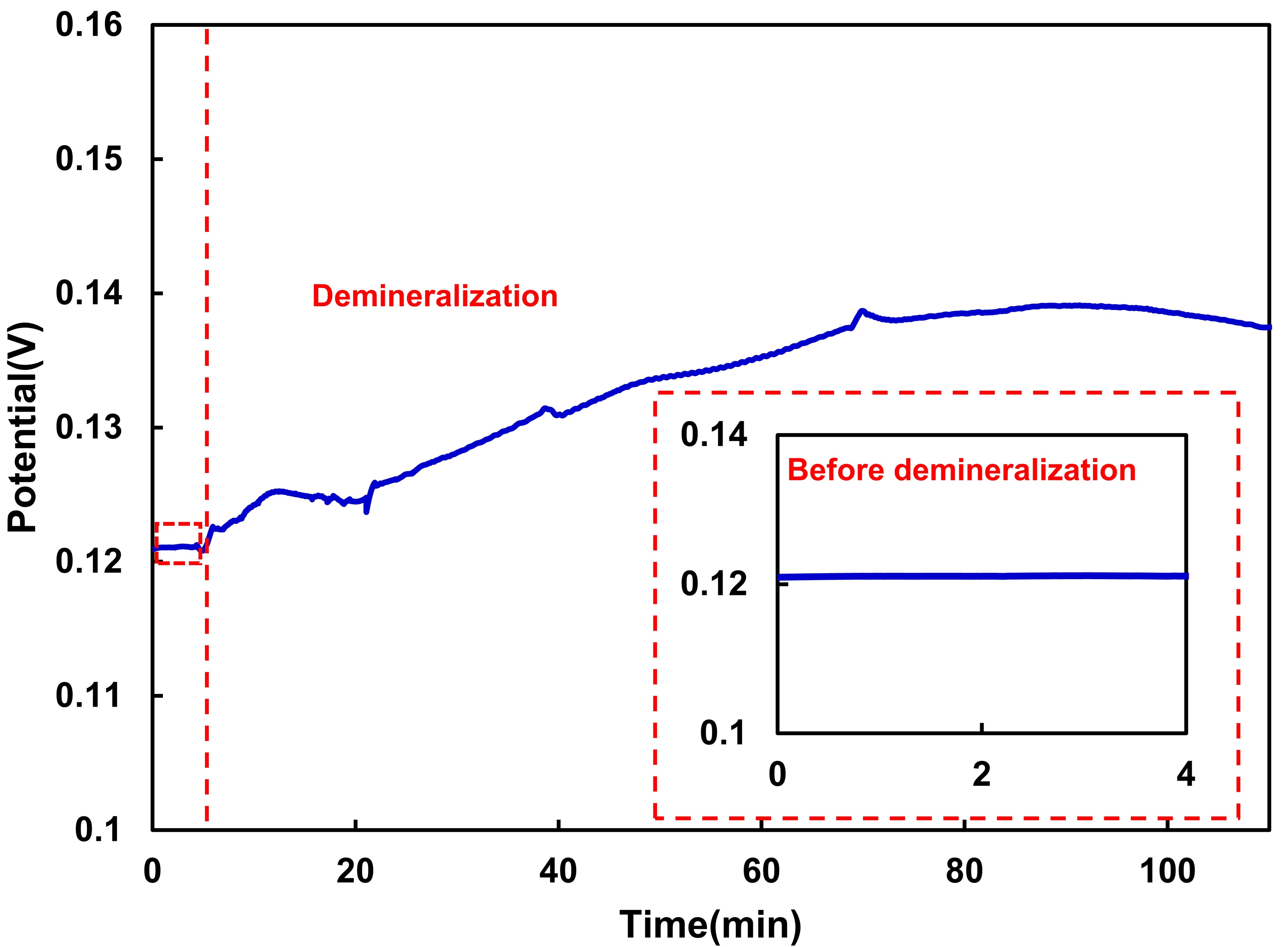


**Figure S18**. The Ca2+ concentration measurement during the demineralization process when a tooth was immersed in an acidic solution (3 mL, pH = 4.3).


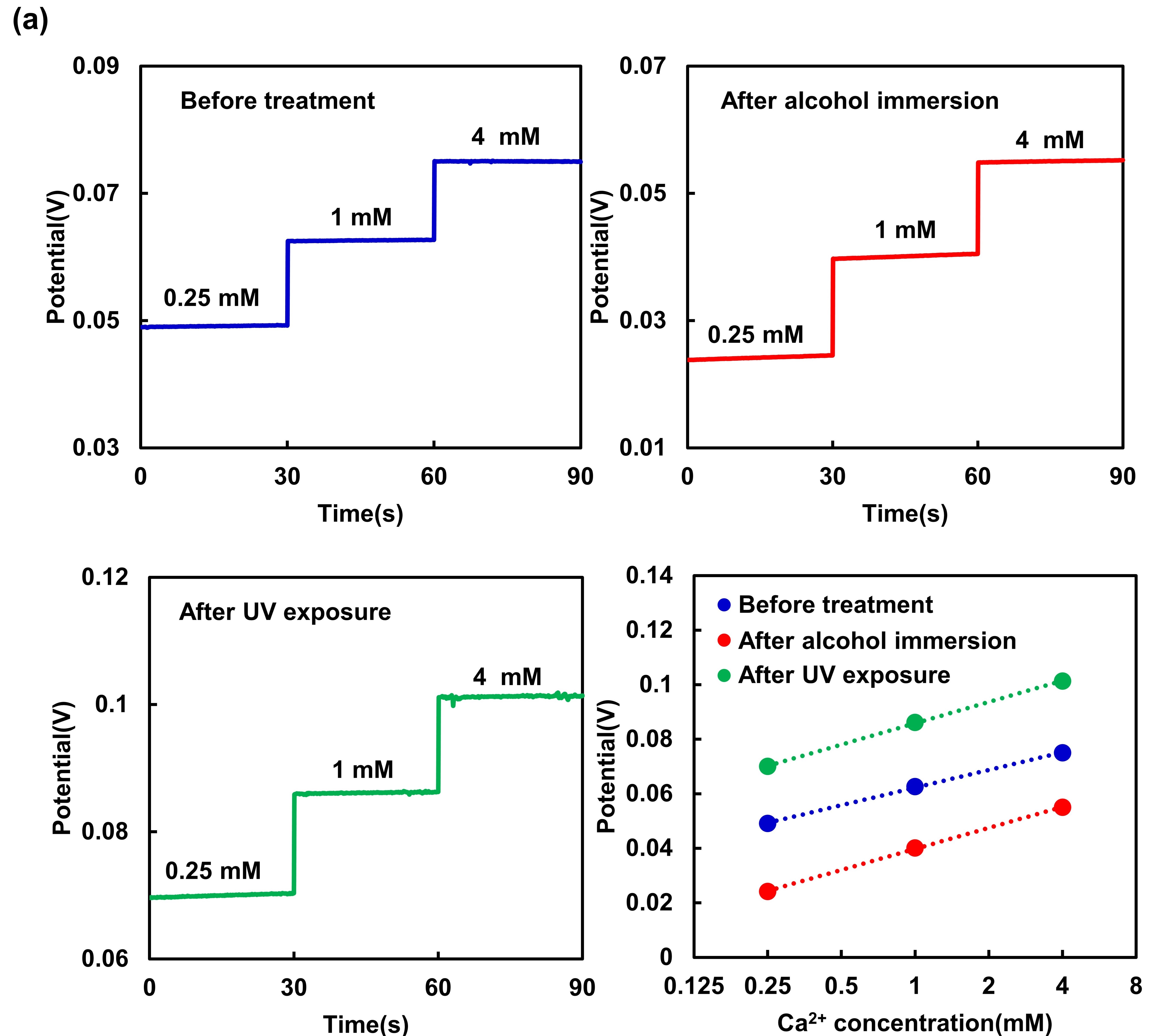

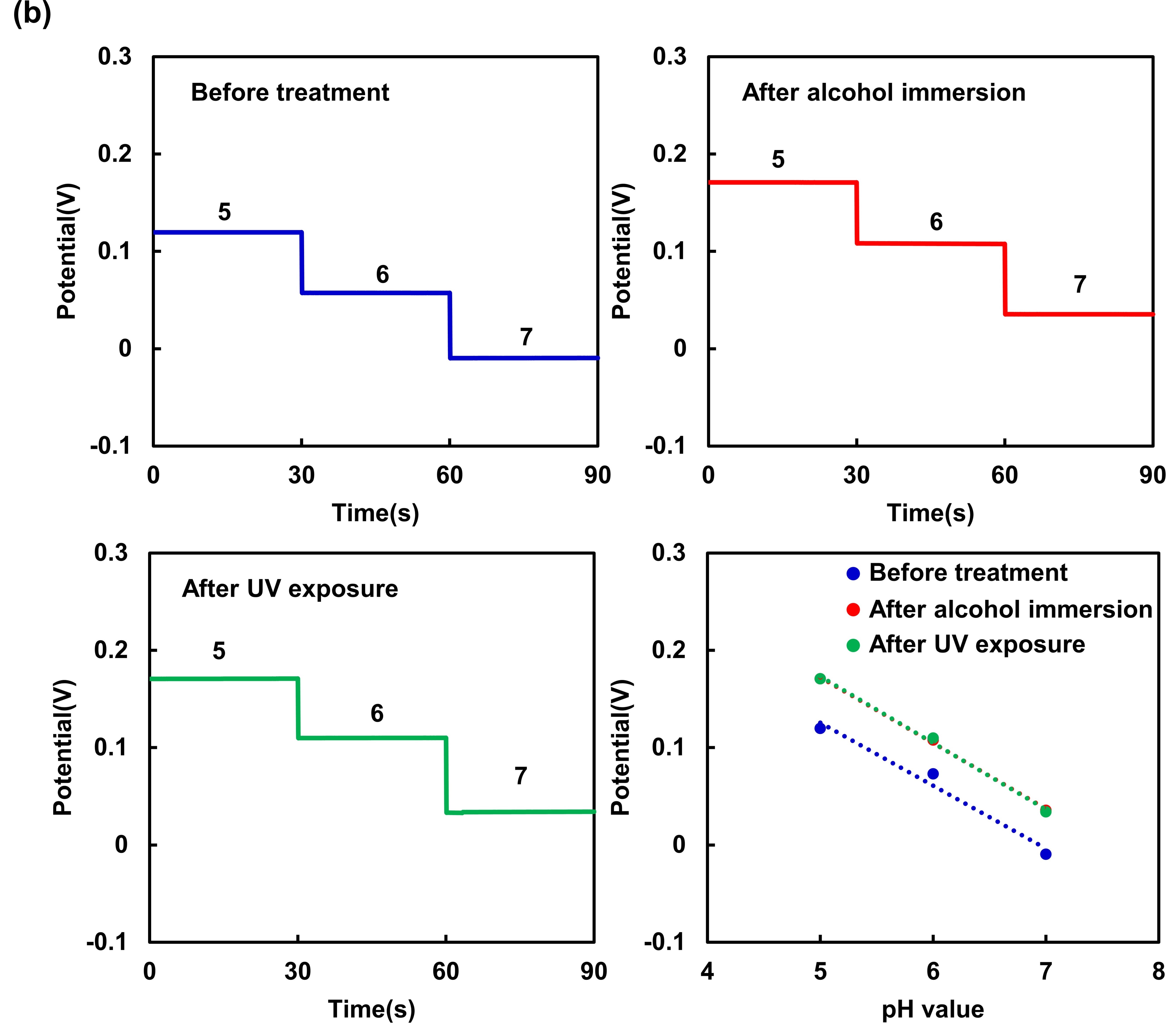


**Figure S19**. The sensing performance of a Ca2+ sensor and a pH sensor disinfected by 75% alcohol immersion and ultraviolet exposure, respectively.


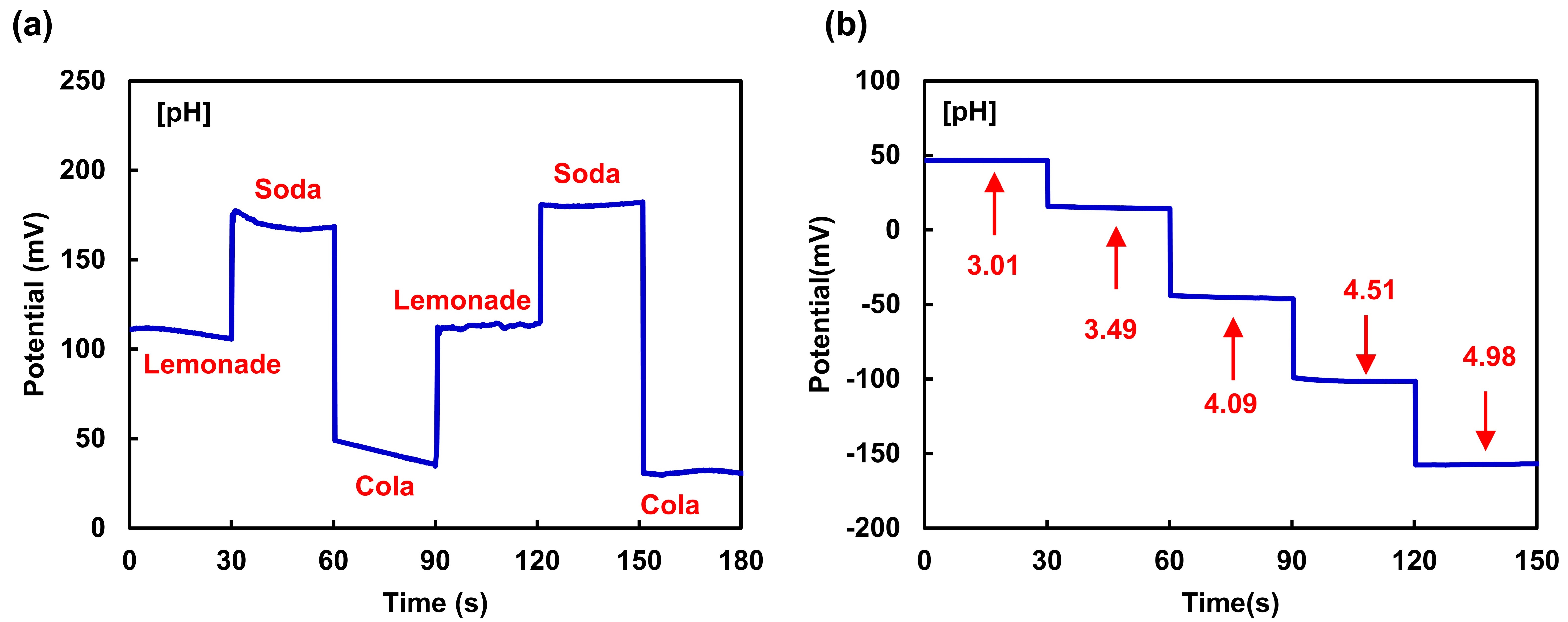


**Figure S20**. *In-vivo*, real-time pH monitoring when drinking different beverages with different acidities. Data were tested with the drink in the mouth for 30 s.


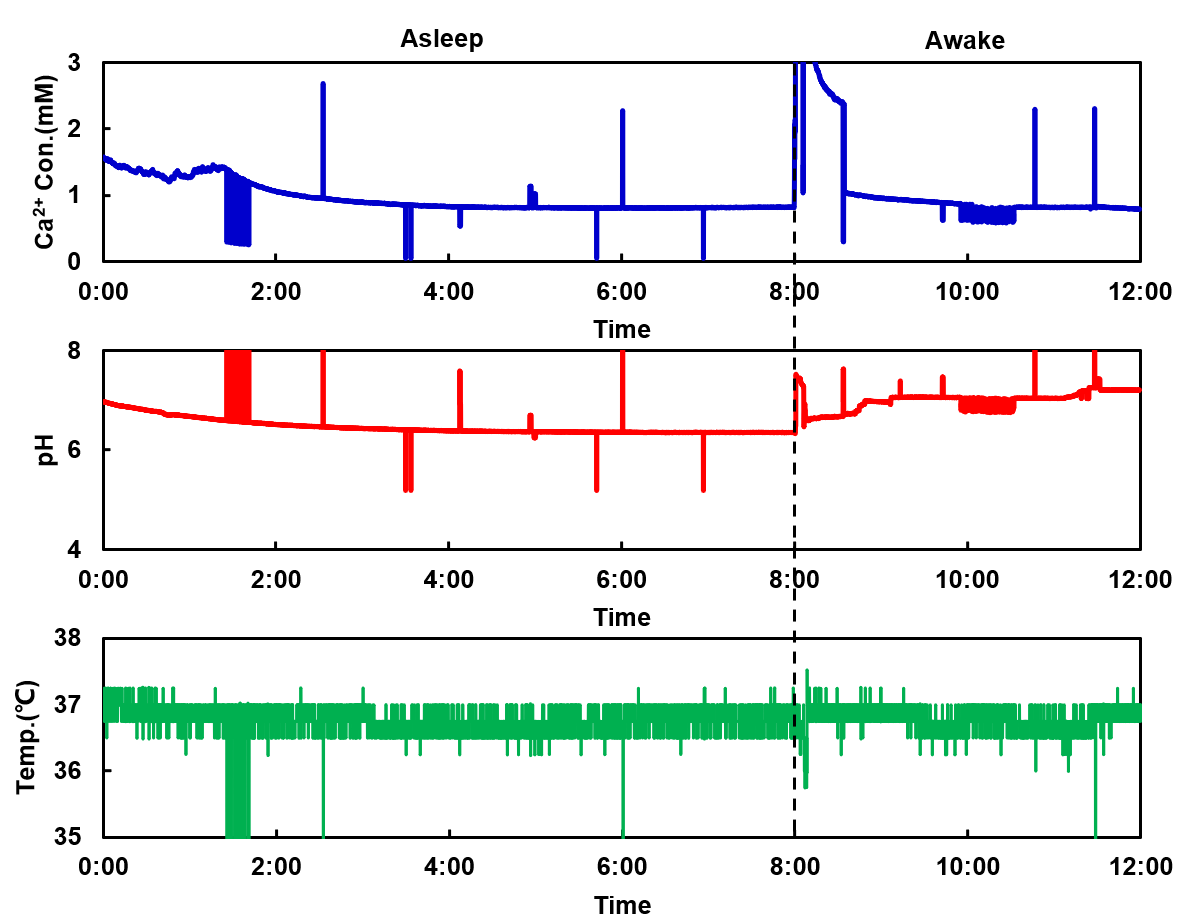


**Figure S21**. Continuous monitoring of intra-oral physiology for 12 hours.


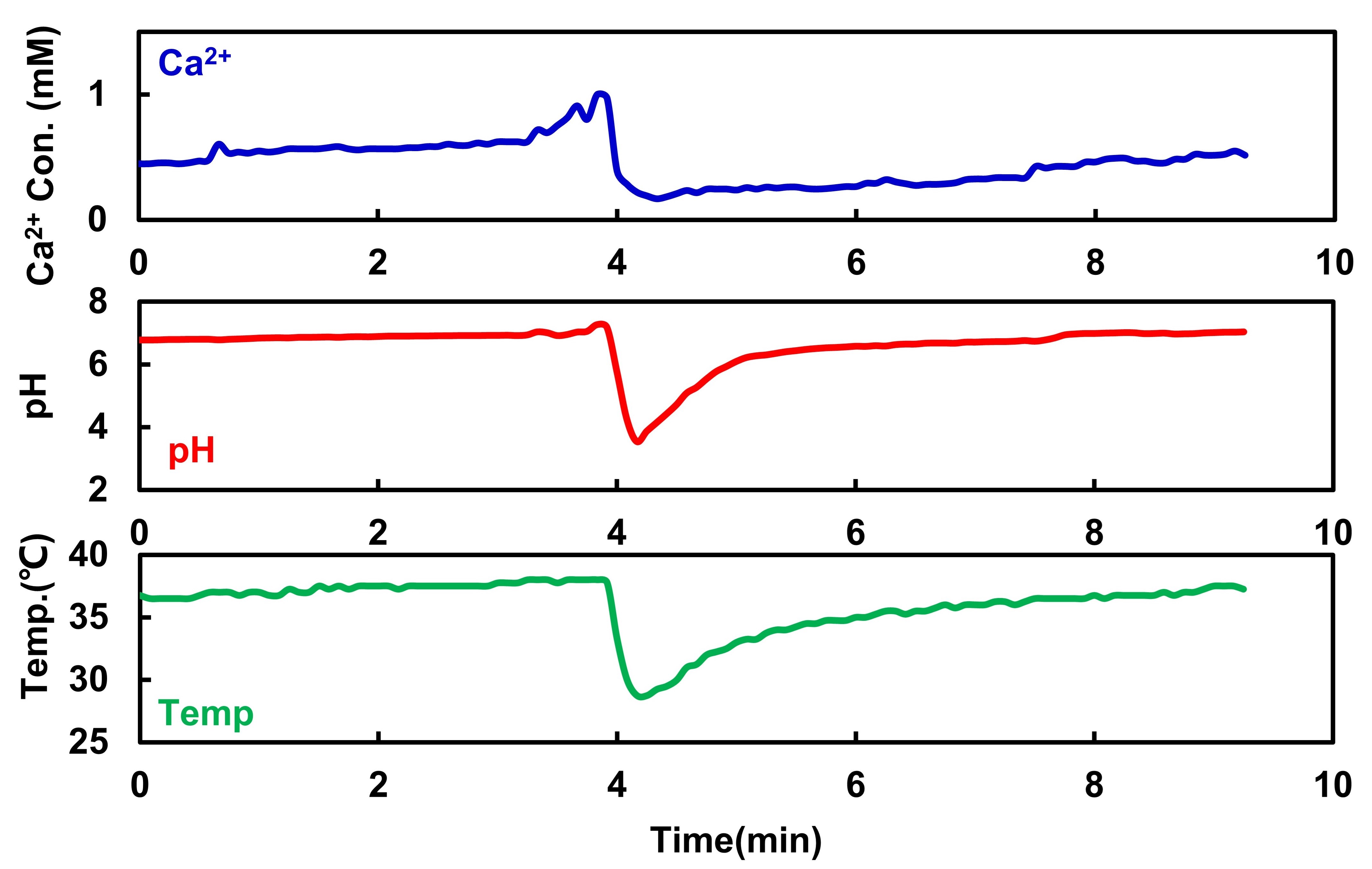


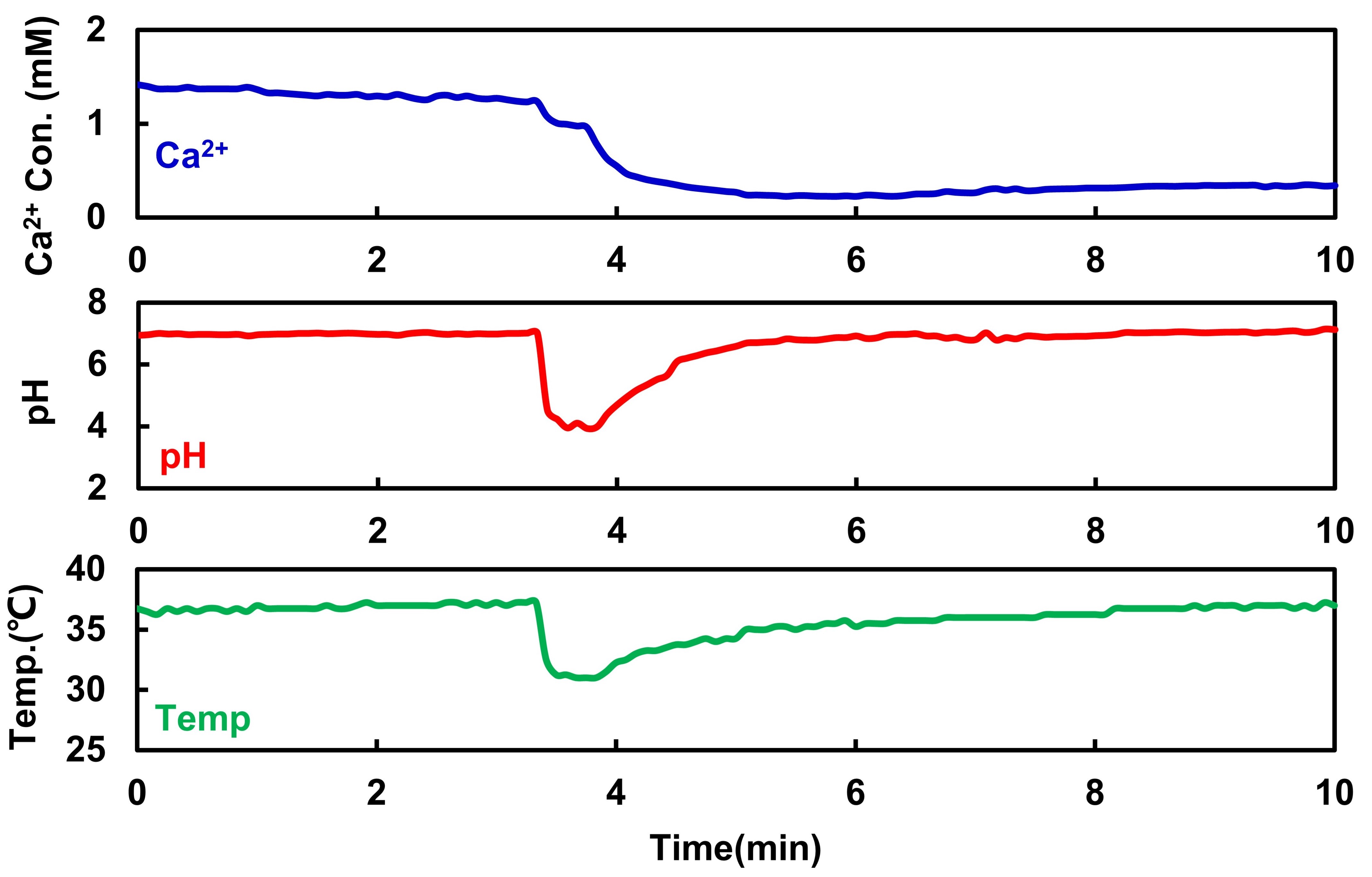


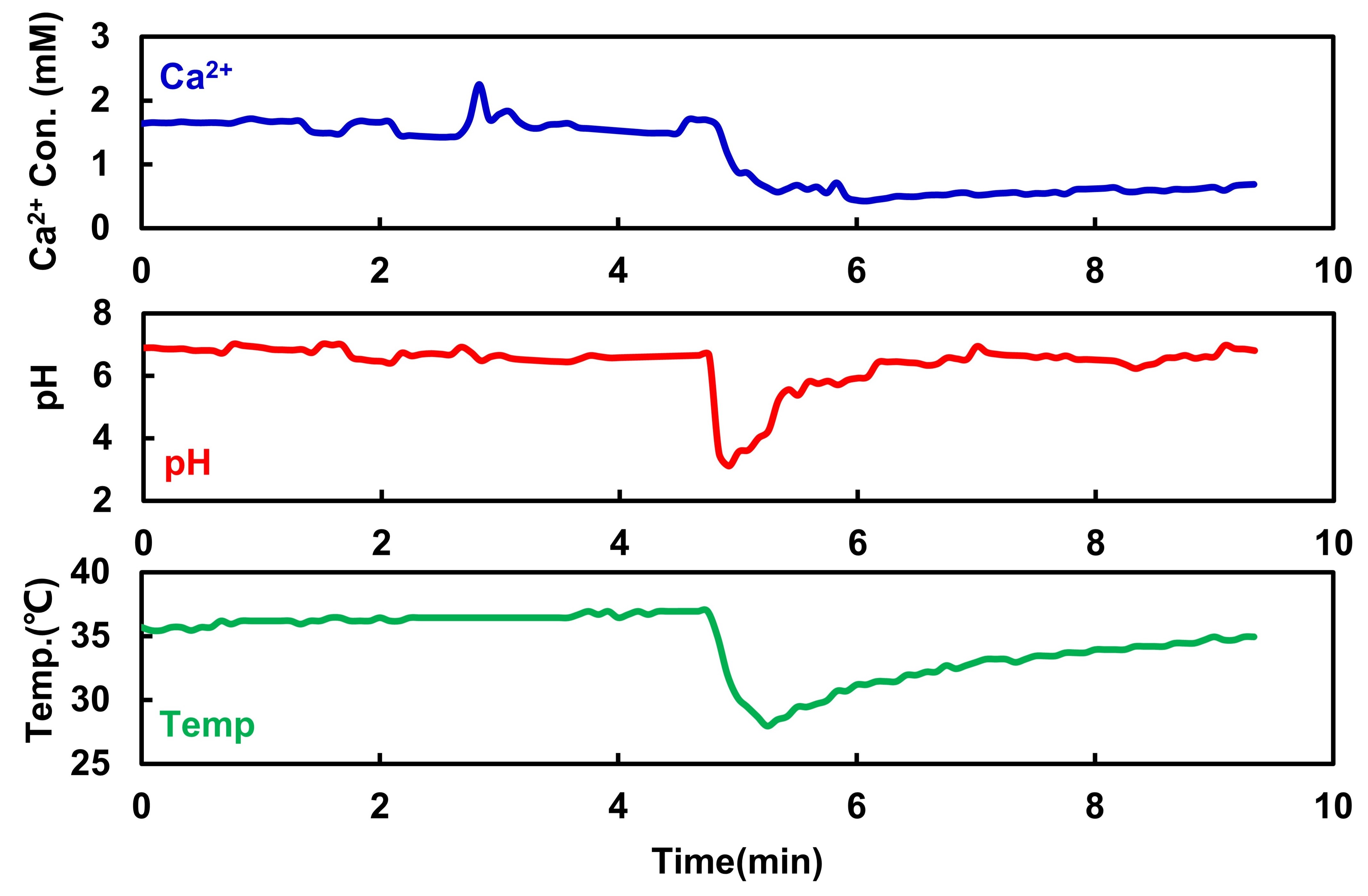


**Figure S22**. Representative sensing results of salivary Ca2+ concentration, pH value, and temperature in 3 dental caries patients after acid stimulation.


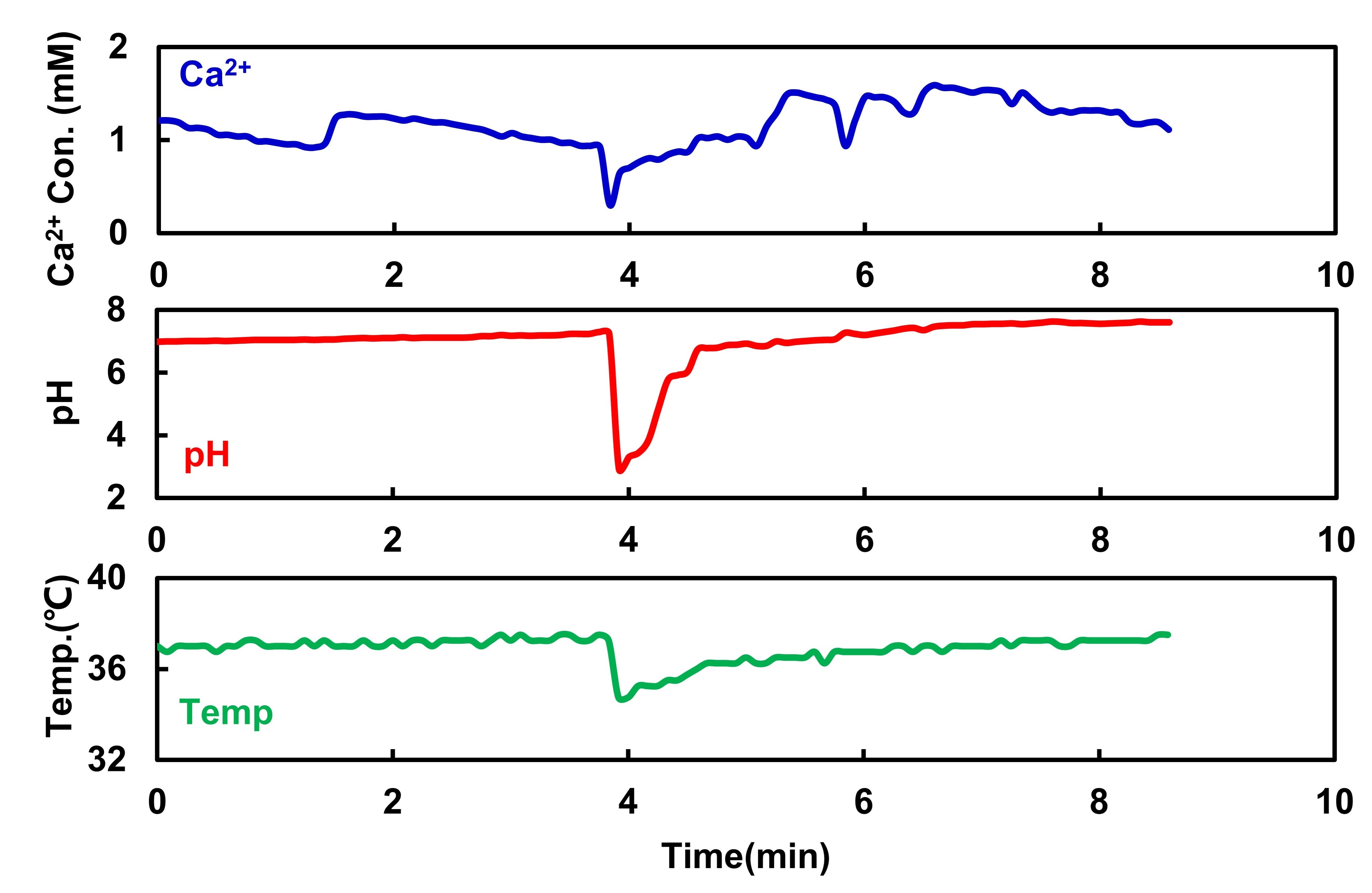


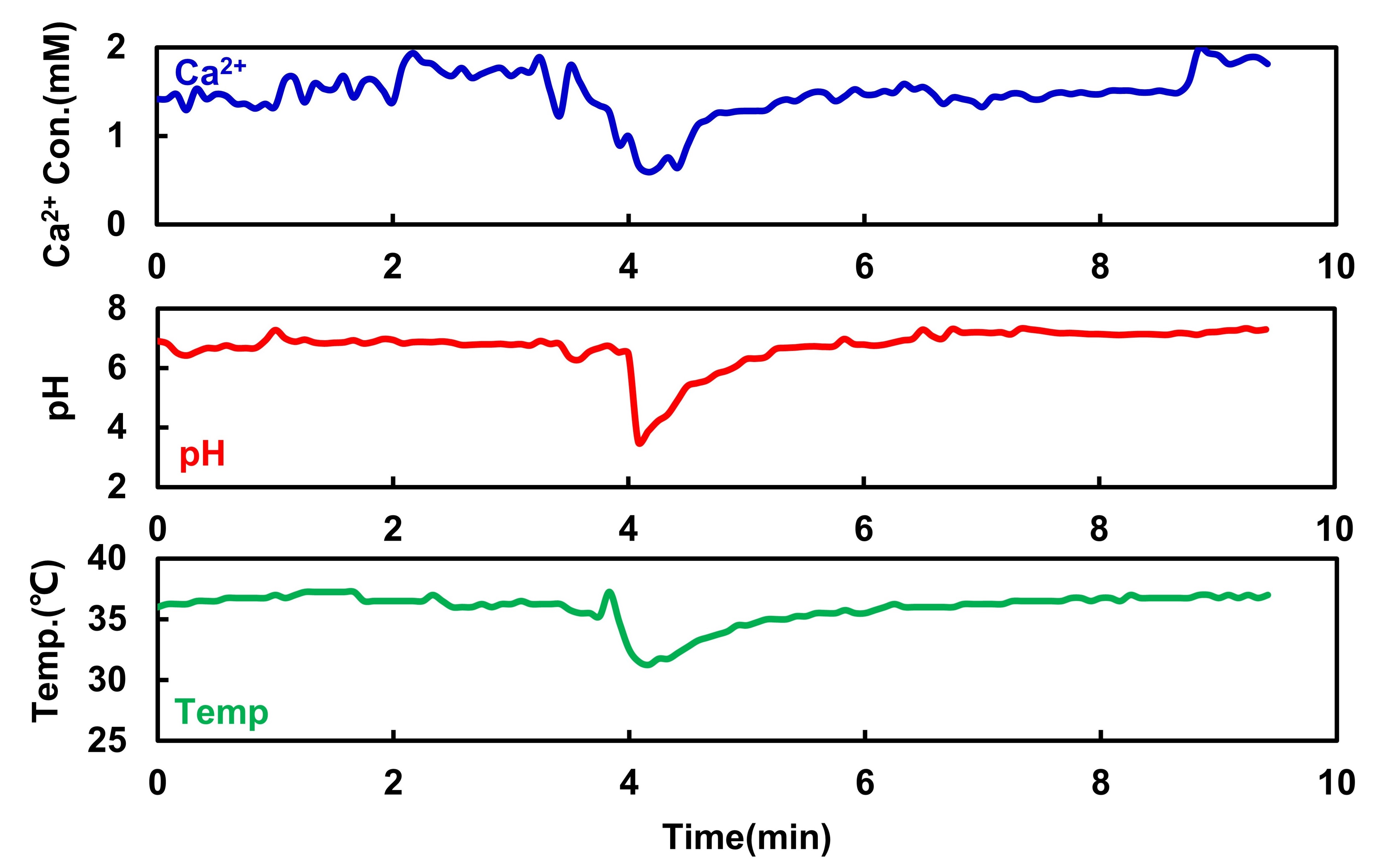


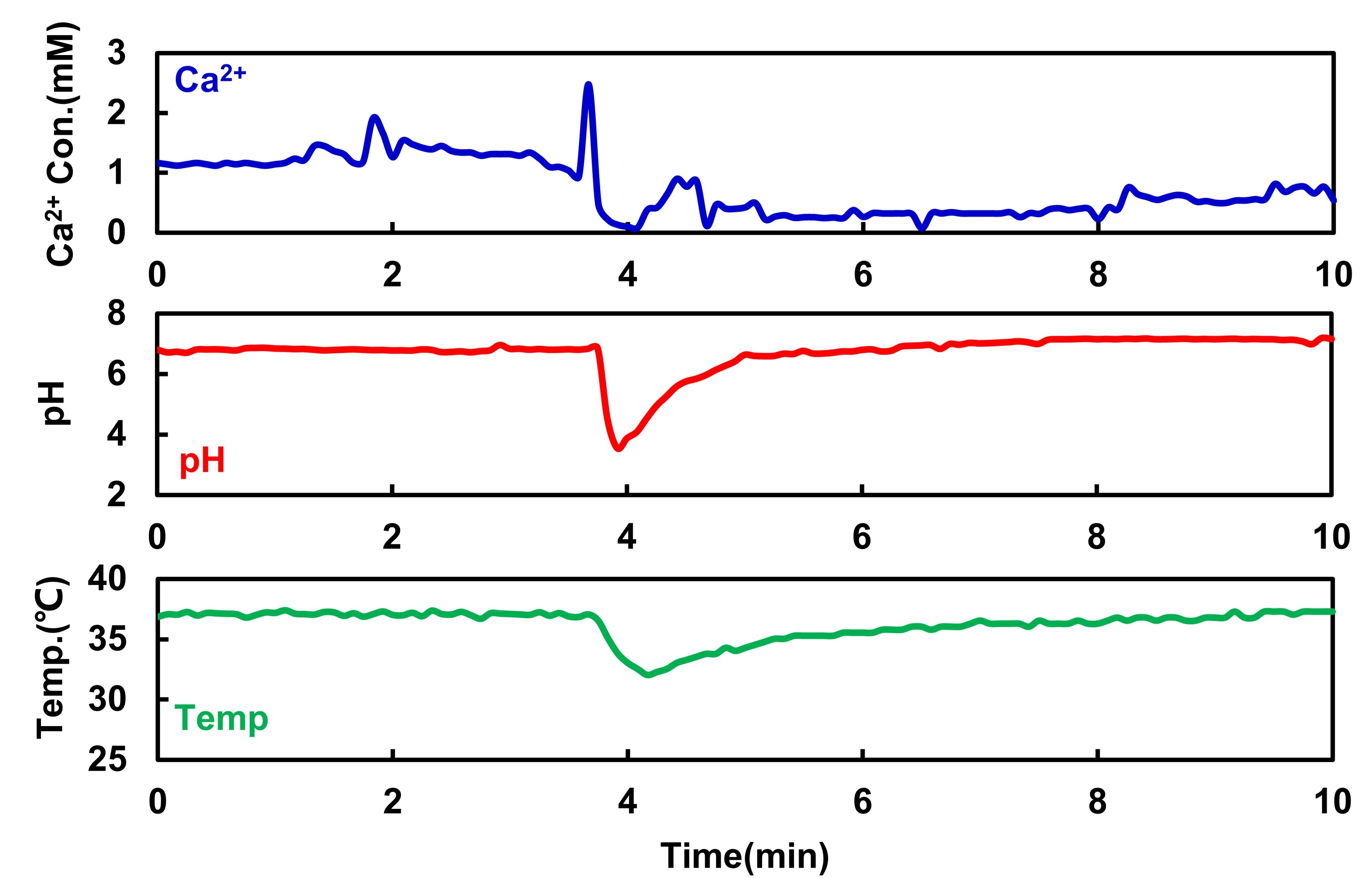


**Figure S23**. Representative sensing results of salivary Ca2+ concentration, pH value, and temperature in 3 caries-free people after acid stimulation.

**Table S1**. Intrinsic pH and measured salivary pH at different time intervals after consumption of different beverages.

| **Beverages** | **Intrinsic pH** | **0 min** | **0.5 min** | **1 min** | **1.5 min** | **2 min** | **3 min** |
| --- | --- | --- | --- | --- | --- | --- | --- |
| Sprite | 3.29 | 3.812 | 4.188 | 4.672 | 5.756 | 6.963 | 7.091 |
| Fanta | 2.51 | 2.672 | 3.353 | 3.963 | 5.826 | 7.014 | 7.043 |
| Pepsi light * | 3.10 | 3.209 | 3.756 | 4.865 | 5.609 | 6.404 | 7.049 |
| Coca Cola | 2.52 | 2.753 | 3.44 | 5.724 | 6.345 | 6.882 | 7.057 |
| NFC orange juice † | 3.90 | 4.451 | 5.047 | 6.131 | 6.812 | 7.022 | 7.127 |
| Reconstituted orange juice | 3.50 | 4.044 | 4.956 | 5.233 | 6.514 | 7.033 | 7.075 |
| Lemonade | 2.85 | 3.242 | 5.472 | 6.358 | 6.882 | 7.007 | 7.033 |

* a caffeine-free cola without sugar.

† a juice not from concentrate.

**Table S2**. Caries risk assessment based on general health, clinical conditions, and salivary buffering capacity.


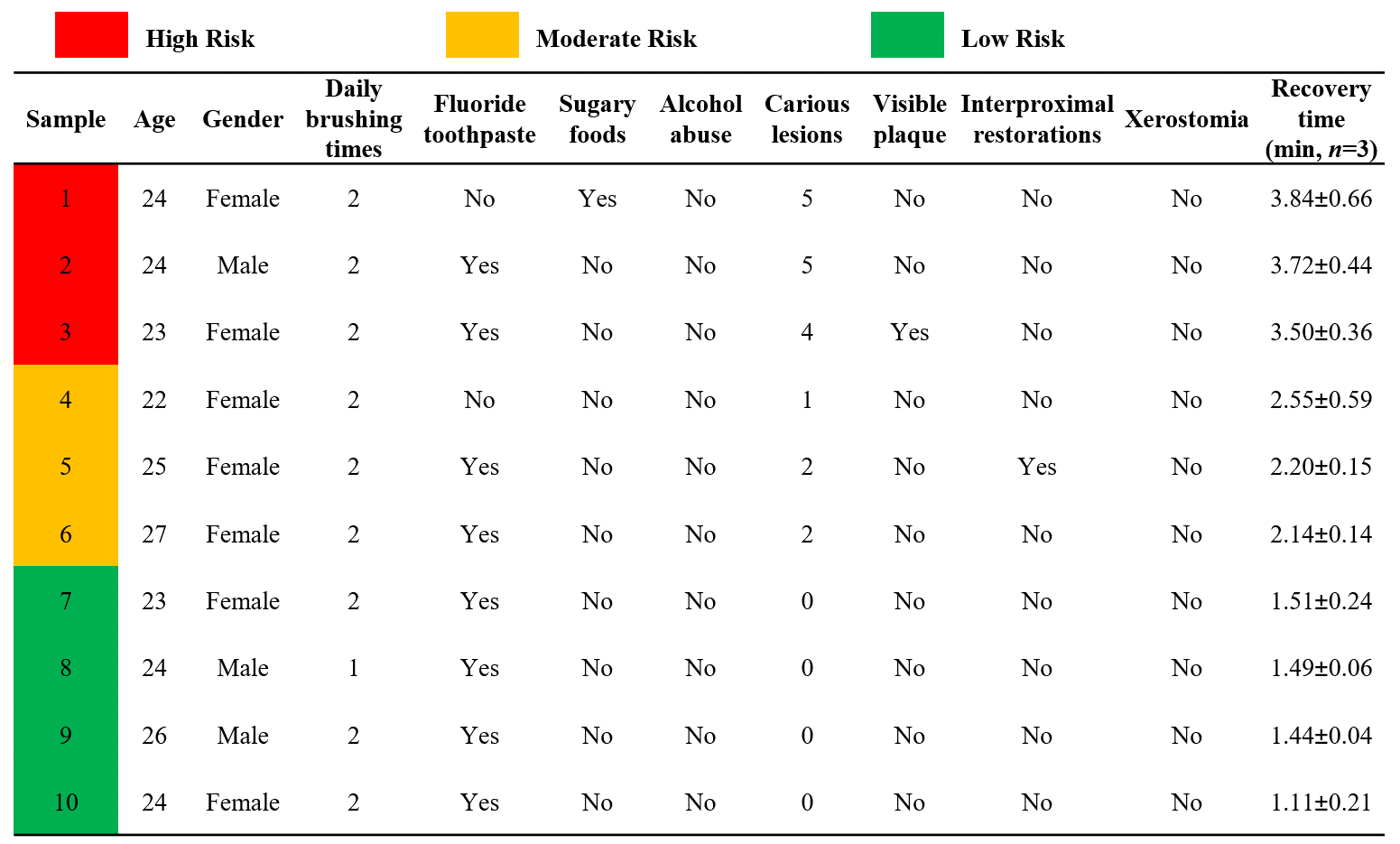

Supplement: Supplementary Materials — Figure S1: SEM images of a planar Au electrode and a AuNP-modified electrode. Figure S2: an image of a flexible sensor attached to teeth surfaces. Figure S3: reversible magnetoelectrical interfaces between different modules using conductive magnets. Figure S4: images of a sensor, a circuit, and two batteries connected in series under extreme bending. Figure S5: an illustration of the circuit design and detailed chip information for the intraoral system. Figure S6: a flexible customized dental brace under stretching, bending, and twisting. Figure S7: the weight of a flexible system and a dental brace embedded with a system. Figure S8: a subject wearing a dental brace integrated with a flexible system. Figure S9: CV curves of electrochemical polymerization of polyaniline with a scan rate of 0.1 V/s. Figure S10: CV curves of a PANI-modified electrode measured at different scan rates from 25 to 300 mV/s. Figure S11: EIS analysis of planar Au electrodes before and after different types of surface modifications. Figure S12: potential stability of a PVB-coated reference electrode and a solid-state Ag/AgCl electrode. Figure S13: reversibility of a Ca2+ sensor with reciprocating concentrations from 0.5 mM to 2 mM. Figure S14: potential stability of a Ca2+ sensor under different pH values. Figure S15: reproducibility of Ca2+ sensors (n = 6) and pH sensors (n = 4). Figure S16: stability of the system for Ca2+ and pH sensing with repeated fluid flushing at a flow rate of 120 mL/min. Figure S17: stability of system encapsulation in artificial saliva. Figure S18: the Ca2+ concentration measurement during the demineralization process when a tooth was immersed in an acidic solution (3 mL, pH = 4.3). Figure S19: the sensing performance of a Ca2+ sensor and a pH sensor disinfected by 75% alcohol immersion and ultraviolet exposure, respectively. Figure S20: in vivo, real-time pH monitoring when drinking different beverages with different acidities. Data were tested with the drink in t [file 9810129.f1.zip › Research-Supplementary Materials.docx]
